# Long term conservation of human metabolic phenotypes and link to heritability

**DOI:** 10.1007/s11306-014-0629-y

**Published:** 2014-02-26

**Authors:** Noha A. Yousri, Gabi Kastenmüller, Christian Gieger, So-Youn Shin, Idil Erte, Cristina Menni, Annette Peters, Christa Meisinger, Robert P. Mohney, Thomas Illig, Jerzy Adamski, Nicole Soranzo, Tim D. Spector, Karsten Suhre

**Affiliations:** 1 Department of Physiology and Biophysics, Weill Cornell Medical College in Qatar, Education City, Qatar Foundation, P.O. Box 24144, Doha, Qatar; 2 Computers and System Engineering Department, Faculty of Engineering, Alexandria University, Alexandria, Egypt; 3 Institute of Bioinformatics and Systems Biology, Helmholtz Zentrum München, German Research Center for Environmental Health, Ingolstädter Landstraße 1, 85764 Neuherberg, Germany; 4 Institute of Genetic Epidemiology, Helmholtz Zentrum München, German Research Center for Environmental Health, Ingolstädter Landstraße 1, 85764 Neuherberg, Germany; 5 Institute of Epidemiology II, Helmholtz Zentrum München, German Research Center for Environmental Health, Ingolstädter Landstraße 1, 85764 Neuherberg, Germany; 6 Metabolon, Inc., Durham, NC USA; 7 Hannover Unified Biobank, Hannover Medical School, Carl-Neuberg-Straße 1, 30625 Hannover, Germany; 8 Institute of Experimental Genetics, Genome Analysis Center, Helmholtz Zentrum München, German Research Center for Environmental Health, Ingolstädter Landstraße 1, 85764 Neuherberg, Germany; 9 Human Genetics, Wellcome Trust Sanger Institute, Hinxton, CB10 1HH UK; 10 Department of Twin Research and Genetic Epidemiology, Kings College London, London, SE1 7EH UK; 11 MRC Integrative Epidemiology Unit, University of Bristol, Bristol, UK

**Keywords:** Metabolomics, Longitudinal study, Heritability, Population study

## Abstract

Changes in an individual’s human metabolic phenotype (metabotype) over time can be indicative of disorder-related modifications. Studies covering several months to a few years have shown that metabolic profiles are often specific for an individual. This “metabolic individuality” and detected changes may contribute to personalized approaches in human health care. However, it is not clear whether such individual metabotypes persist over longer time periods. Here we investigate the conservation of metabotypes characterized by 212 different metabolites of 818 participants from the Cooperative Health Research in the Region of Augsburg; Germany population, taken within a 7-year time interval. For replication, we used paired samples from 83 non-related individuals from the TwinsUK study. Results indicated that over 40 % of all study participants could be uniquely identified after 7 years based on their metabolic profiles alone. Moreover, 95 % of the study participants showed a high degree of metabotype conservation (>70 %) whereas the remaining 5 % displayed major changes in their metabolic profiles over time. These latter individuals were likely to have undergone important biochemical changes between the two time points. We further show that metabolite conservation was positively associated with heritability (rank correlation 0.74), although there were some notable exceptions. Our results suggest that monitoring changes in metabotypes over several years can trace changes in health status and may provide indications for disease onset. Moreover, our study findings provide a general reference for metabotype conservation over longer time periods that can be used in biomarker discovery studies.

**Electronic supplementary material:**

The online version of this article (doi:10.1007/s11306-014-0629-y) contains supplementary material, which is available to authorized users.

## Introduction

The ‘omics’ field has facilitated measuring thousands of biological entities (e.g., genes, proteins, mRNA transcripts, and metabolites) with the aim of detecting correlations between them or their possible association to a disease phenotype. Metabolomics aims at measuring the concentrations of small molecules or metabolites in a given biological fluid such as plasma, urine, saliva, and breath (Fiehn 2002; Lindon et al. 2006; Martinez-Lozano Sinues et al. 2013; Zhang et al. 2012). Today, with advances in biotechnology, modern mass spectrometry has allowed for comprehensive measurement of many endogenous metabolites in a biological fluid. Non-targeted metabolomics approaches enable the measurement of several hundred to a thousand or more metabolites from a variety of different metabolic pathways (Kettunen et al. 2012; Suhre et al. 2011a). Such technologies facilitate detailed metabolic profiles to an extent that has not been accessible before. The metabolites that are measured for each individual characterize the human metabolic phenotype (or “metabotype”), which is defined as “a probabilistic multi-parametric description of an organism in a given physiological state based on the analysis of its cell types, biofluids or tissues” (Gavaghan et al. 2000). Human metabotypes thus represent a comprehensive readout of the biological state of the human body and have been associated with a number of human disorders (Amara and Standaert 2013; Fiehn 2002; Halama et al. 2013; Holmes et al. 2008a, b; Kaddurah-Daouk et al. 2008; Langley et al. 2013; Suhre et al. 2010, 2011a; Wang et al. 2013).

Metabolomics provides information about the joint effects of both environmental and lifestyle factors (such as dietary patterns) and genomics. Changes in the metabolome can be described by the activity of genes, enzymes, and proteins that can be associated with conventional or new target therapies (Corona et al. 2012). This application is relevant for identifying disease biomarkers from metabolites, as can be possible in diabetes, obesity, and cancers (Griffin and Shockcor 2004; Suhre et al. 2010; Menni et al. 2013) where alterations of metabolite concentrations can provide early evidence of disease onset (Assfalg et al. 2008). Thus, pharmaco-metabolomics, which combines metabolite profiling and chemometrics to model and predict the efficacy of drug intervention in individuals, has benefited from this technology (Corona et al. 2012). The field of nutrigenomics, in which appropriate dietary choices are sought to avoid metabolic imbalances leading to disease, presents another motivation for profiling individual metabolites (German et al. 2004). However, such fields mainly depend on the “individuality” or “uniqueness” of human metabolic profiles because treatment, diet, or drug selection is specific for each individual based on a specific profile and one dietary plan can work optimally for an individual but may predispose others to disease (German et al. 2004).

Although a large day-to-day variability has been shown in individual metabotypes (Krug et al. 2012; Dallmann et al. 2012), the metabotype tends to be characteristic of each person. Both genetic differences and environmental factors play a role in such “individuality” (Assfalg et al. 2008; Bernini et al. 2009). The variability in an individual could be due to “diurnal changes (Walsh et al. 2006), hormonal status (Bollard et al. 2005), and stage in the menstrual cycle for women of reproductive age (Wallace et al. 2010)” while differences that characterize individual metabotypes could be due to factors such as “gender, age, and adiposity (Gu et al. 2009; Kochhar et al. 2006; Rasmussen et al. 2011; Winnike et al. 2009) or from less well-characterized habitual dietary patterns and other environmental and cultural influences (Lenz et al. 2003; Holmes et al. 2008a, b)” (Fave et al. 2011). (Heinzmann et al. 2012) indicated that, regardless of dietary patterns, each individual has a core metabolic fingerprint, influenced by a combination of many factors such as host metabolism, gut microbiota composition, dietary habits, physical activity, and body composition. Several studies have considered this individuality; for example, (Sampson et al. 2013) studied within-subject and between-subject variability and identified metabolites with high within-subject variability that can be used to distinguish among individuals/metabotypes.

Rather than being only characteristic to each individual at any given time point, metabotypes should be monitored for their persistence to a specific individual, or “conservation” over time. Assfalg et al. (2008), and Bernini et al. (2009) observed clusters of metabotypes of the same individual taken at different time points, which resulted from higher variability in inter-individual profiles compared to intra-individual profiles. Martinez-Lozano Sinues et al. (2013) observed the persistence of individual signatures over time in some biofluids, such as breath. However, the time intervals considered by such studies tend to be relatively short. For example, (Saude et al. 2007) studied the variability in human urine over of a period of 1 month, and (Nicholson et al. 2011) investigated the stable components in plasma and urine metabolites over a period of 4 months. In general, studies have monitored metabolic profiles for short periods, ranging from a few months and up to 2 or 3 years. However, the persistence of the individuality of metabotypes over longer time periods remains unexplored.

Metabolites shape the persistence of metabotype individuality over time, and identifying the degree of stability of a metabolite over long periods is thus necessary, in particular for biomarker discovery studies; biomarker concentration should not vary too much over the short term within an individual because such variation would undermine the predictive association from a single sample (Nicholson et al. 2011). In addition, such markers should not be completely heritable if environmental factors significantly influence disease risk. The predictive power of a biomarker has been speculated to be nested within the metabolite’s longitudinally stable component, yielding an intriguing question about the stability component of a metabolite versus its heritability component (Nicholson et al. 2011). However, as noted, only short periods have been used for such studies, and the challenge is that (Nicholson et al. 2011) expected a gradual smooth decay in stable behavior of metabolites with an increasing time scale from months to years.

We study the conservation of individual metabolic profiles or metabotypes over a long term while considering the effect of metabolite conservation (also referred to as stability). Note that we use the term “longitudinal study” in the narrow sense of a study that considers only two points in time for every individual. We also address the question of whether metabolite stability decays over time by studying the conservation of metabolites. We further examine the relation of heritability estimates to metabolite conservation, to understand the metabolome. While previous studies covered time spans on the order of months or only 2 or 3 years, the present study is by far the first to expand research into a longitudinal study of metabotypes/metabolites to time spans of up to 7 years with information on metabolite heritability. Pearson correlation was used in this study to measure correlation of a metabolic profile to itself (intra-correlations), as well as correlations to other profiles (inter-correlations) at the two time points. Being also done for metabolites, testing the difference in distributions between intra-correlations and inter-correlations is an initial step of investigating conservation behavior. A measure derived from these correlations is the conservation index (see Sect. 2), which we have used to show that over an interval of 7 years, a large fraction of the population displays a high degree of metabotype conservation that correlates with metabotype heritability and that drastic changes in metabotype occur in only a small fraction of study participants. These latter differences may be indicative of important physiological and potentially disease-related changes.

## Materials and methods

### Study population

Cooperative Health Research in the Region of Augsburg (KORA) is an epidemiological research cohort with participants randomly selected from the general population in the region of Augsburg in Southern Germany (Holle et al. 2005). Here we use samples and data that were collected during the fourth survey (KORA S4) between 1999 and 2001 and from a follow-up study (KORA F4) that was conducted 7 years later between 2006 and 2008. Extensive examinations and phenotyping using standardized protocols have been applied and are described in detail elsewhere (Wichmann et al. 2005 and references therein). Of 3,080 individuals who attended both studies, 818 who had metabolite profiles and other covariates measured at both baseline and follow-up visits were included in this analysis. These participants were between the ages of 54 and 75 years at baseline with an equal distribution of males and females. KORA is used here as the discovery study. For replication, we used data and samples from a subset of 83 unrelated participants of the TwinsUK cohort. TwinsUK is a British adult-twin registry with predominantly female participants. Samples not suitable for this project from TwinsUK, such as data from a second twin or having a too short time span between two samplings, were removed. Study participants were enrolled from the general population through national media campaigns and were shown to be comparable to age-matched population singletons in terms of disease-related and lifestyle characteristics (Andrew et al. 2001). The samples used in this study were derived from participants ages 30–75 (median 58 years) and are all from females (because most TwinsUK participants are female). Multiple samples of TwinsUK participants were collected at varying time intervals. Here we selected samples that were collected in variable ranges, with a minimum of 1 year and a maximum of 13 years apart (mean 8 years; 75 % of the data points have a range of 6–13 years; histogram is given in Supplemental Figure 1). For both studies, all participants gave written informed consent. The studies were approved by the local ethics committees, the Bayerische Landesärztekammer for KORA and Guy’s and St. Thomas’ Hospital Ethics Committee for TwinsUK.

### Blood sampling

Blood for KORA F4 was drawn between 8:00 a.m. and 10:30 a.m. after 10 h fasting. Material was drawn into serum gel tubes, gently inverted twice, rested for 30 min at room temperature to obtain complete coagulation, and then centrifuged for 10 min at 2,750×*g*. Serum was divided into aliquots and kept for a maximum of 6 h at 4 °C, after which it was stored at −80 °C until analysis. A similar blood draw protocol was used in KORA S4 (Rathmann et al. 2003). For the TwinsUK study, blood samples were taken after at least 6 h of fasting. The samples were immediately inverted three times, followed by 40 min of resting at 4 °C to obtain complete coagulation. The samples were then centrifuged for 10 min at 2,000×*g*. Serum was removed from the centrifuged brown-topped tubes as the top, yellow, translucent layer of liquid. Aliquots were stored at −45 °C until sampling.

### Metabolomics measurements

Metabolic profiling was done on serum using ultrahigh-performance liquid-phase chromatography and gas-chromatography separation, coupled with tandem mass spectrometry (UHPLC/MS/MS2 and GC/MS, respectively) at Metabolon, Inc. (Durham, NC, USA) using established procedures and technology (Evans et al. 2009; Suhre et al. 2011b). Briefly, Metabolon is a commercial supplier of metabolic analyses that developed a platform integrating chemical analysis, including the identification and relative quantification, data reduction, and quality-assurance components of the process. Samples are submitted to three analyses: to positive- and negative-mode UHPLC/MS/MS2 and to GC/MS. The UHPLC injections were optimized for basic and acidic species. The resulting MS/MS2 data were searched against a standard library generated by Metabolon that included retention time, molecular mass-to-charge ratio (*m*/*z*), and preferred adducts and in-source fragments as well as their associated MS/MS spectra for all molecules in the library. The library allowed for the identification of the experimentally detected molecules on the basis of a multi-parameter match without the need for additional analyses. RSD (relative standard deviation) was determined using repeated measurements of the technical replicates in pooled samples. Supplemental Table 1 gives details of measurements associated with each metabolite. Metabolomics measurements for KORA S4, KORA F4, and TwinsUK were performed in separate batches at Metabolon. Metabolites with more than 20 % missing values or that were detected only in either the S4 or the F4 samples were removed, resulting in a dataset of 212 metabolite levels for 818 participants in KORA and 203 metabolites for 83 participants in TwinsUK. A total of 135 metabolites were common to the KORA and the TwinsUK datasets. Missing data were imputed to the average over all valid observations of that metabolite at the respective time point. Metabolite concentrations were z-scored normalized over all samples.

### Statistical analysis

Analysis was done using the R package (2.15.2). The following definitions are used throughout this paper: For an individual, the term *metabotype* is used to refer to the set (or vector) of metabolite concentrations over the entire set of metabolites. Time points (t_1_ or t_2_) refer to the first and second data point in each study, i.e., in KORA, *t*_1_ refers to data from the initial S4 survey and *t*_2_ to the F4 follow-up. The term *metabotype* *correlation* refers to the Pearson correlation between the metabolite profiles of two individuals *ind*_*i*_ and *ind*_*j*_ from a cohort *C* (KORA or TwinsUK) at two time points, written as *r*(*ind*_*i*_^*C*^(*t*_1_), *ind*_*j*_^*C*^(*t*_2_)). *Longitudinal metabotype intra*-*correlation* refers to the correlation of a metabotype of individual *i* at the first time point to that of the same individual at the second time point and is denoted as *r*(*ind*_*i*_^*C*^(*t*_1_), *ind*_*i*_^*C*^(*t*_2_)). *Longitudinal metabotype inter*-*correlation* refers to the correlation of a metabotype of individual *i* at the first time point to that of a different individual *j* at the second time point and is denoted as *r*(*ind*_*i*_^*C*^(*t*_1_), *ind*_*j*_^*C*^(*t*_2_))*. Metabolite correlation* refers to the Pearson correlation between two metabolite concentration vectors *met*_*k*_^*C*^(*t*_*1*_) and *met*_*l*_^*C*^(*t*_*2*_) of metabolites *k* and *l* at time points *t*_*1*_ and *t*_*2*_ and is denoted as *r*(*met*_*k*_^*C*^(*t*_1_), *met*_*l*_^*C*^(*t*_2_)). *Weighted metabotype correlation* refers to the Pearson correlation between two metabotypes at two time points using metabolite correlations as weights, calculated using the following formula:

$$r_{w} \left( {x,y} \right) = \frac{{cov_{w} \left( {x,y} \right)}}{{\sqrt {cov_{w} \left( {x,x} \right) \cdot cov_{w} \left( {y,y} \right)} }}$$

$$cov_{w} \left( {x,y} \right) = \mathop \sum \limits_{k = 1}^{M}
(x_{k} w_{k} - \overline{x}_{w} )(y_{k} w_{k} - \overline{y}_{w}
)$$

$$\overline{x}_{w} = 1/M\mathop \sum \limits_{k = 1}^{M} x_{k} w_{k}$$

where *x* represents a metabolite concentration vector and *M* is the number of metabolites. The weight is calculated as *w*_*k*_ = *r*(*met*_*k*_^*C*^(*t*_1_), *met*_*k*_^*C*^(*t*_2_)). The terms *weighted longitudinal metabotype inter*- and *intra correlations* are used as for the normal correlations defined above, but using weighted correlations.

The *conservation index* is used for both metabotypes and metabolites. The *metabotype conservation index* of an individual *i* is defined as the relative rank of the longitudinal metabotype intra-correlation of that individual with respect to all longitudinal metabotype inter-correlations of that individual with all other individuals from the same study cohort. To calculate this index, the intra-correlations are converted to ranks to measure a metabotype’s or metabolite’s similarity to itself when compared to its similarity to other metabotypes or metabolites. It is calculated as 1 − ((*rank*(*i*) − 1)/(*N* − 1)), where N is number of metabotypes. This index quantifies the comparison of intra-correlations to inter-correlations, yielding a value in the range [0,1]. The metabolite conservation index of a metabolite is calculated in the same manner as the metabotype conservation index by replacing vectors of metabotypes with metabolite concentration vectors. A value of 1 indicates a fully conserved metabotype or metabolite. For example, in a 3-subjects set (A, B, C) (N = 3), metabotype A has a correlation of 0.5 to itself after 7 years, and a correlation of 0.6 and 0.7 to B and C, respectively, after 7 years. Thus, its similarity to itself (0.5) is ranked third among all the other similarities to other metabotypes, and its conservation index is 0. If B has a correlation of 0.8 to itself and 0.5 to C, then its self-correlation is ranked first among correlations to others, meaning that it is fully conserved, thus resulting in a conservation index of 1. The *weighted metabotype conservation index* is defined similarly using weighted metabotype correlation.

### Heritability estimates

Heritability estimates (h) were obtained from previous work based on a large cross-sectional metabolomics dataset from the TwinsUK study using the Metabolon platform (Shin et al., submitted manuscript). A total of 212 metabolites overlapped between the metabolite sets used in that study and the present work. Briefly, heritability was computed using monozygotic and dizygotic twin pairs under the ACE [additive genetic effects (A), shared family environment (C), and unique environment (E)] model (Zyphur et al. 2013), which models trait variance as a function of additive genetics, common environment, and unique environment and/or error effects. The narrow-sense heritability was inferred from the proportion of the total variance explained by estimated additive genetic effect. Calculations were carried out using maximum likelihood methods implemented in OpenMx software (Boker and Neale 2011) while adjusting for age, gender, and batch effects.

### Association with age, gender, and BMI

To estimate the impact of age, gender, and BMI, metabolite levels at KORA S4 were modeled using multi-linear regression in R, with cofactors gender, age, and BMI [R code: lm (metabolites ~ age + gender + BMI)]. The 15 most strongly phenotype-associated metabolites were selected for visualization.

### Principal component analysis (PCA)

R function prcomp was used to obtain the principal components.

## Results

### Metabolic profiles of the same individual taken at time points 7 years apart correlate

**Fig. 1:**
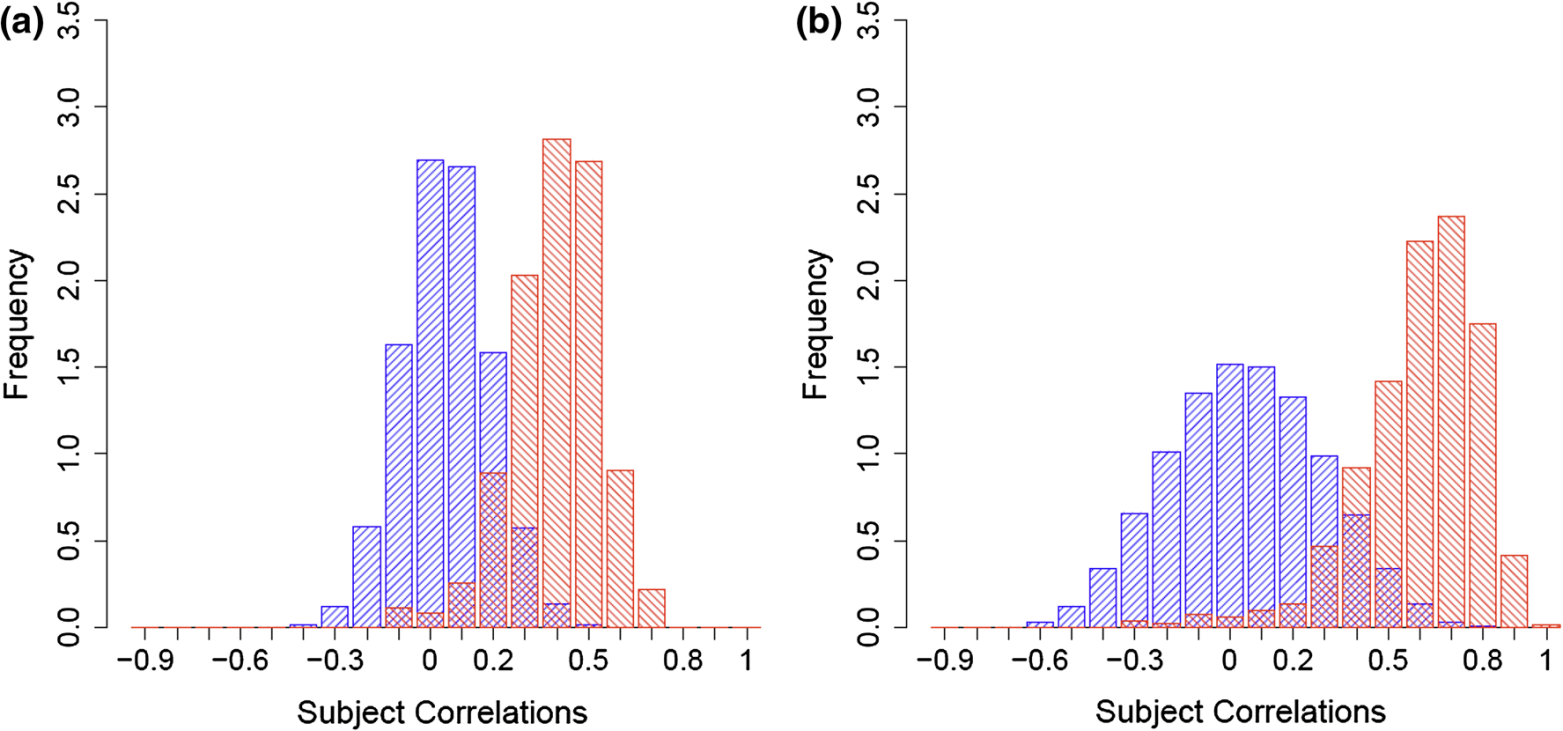
Metabotype pairwise longitudinal inter correlations versus intra correlations distributions between KORA S4 and F4. **a** Pearson correlation of the metabolite levels between two time points for the same individual, or intra-correlations [median is 0.35 (*red histogram*)] and for pairwise inter correlations [median is −0.0012 (*blue histogram*)]. **b** As in **a**, but using metabolite correlations as weights to metabotype correlations [medians are 0.58 for intra-correlations (*red*) and −0.0018 for pairwise inter-correlations (*blue*)]

Pearson correlation was used to calculate intra- and inter-correlations between the metabotypes of individuals at two time points, designated as “longitudinal” intra- and inter-correlations (see “Sect. 2”). These values were calculated based on 818 individual metabotypes using 212 metabolites studied at the two time points S4 and F4 for the KORA cohort. The distributions of the pairwise longitudinal inter- and intra-correlations for KORA are shown in Fig. 1a. The median of the longitudinal intra-correlations was 0.35 and significantly different from zero (*p* < 2.2 × 10^−16^), but there was no observable correlation among the metabotypes of different individuals (longitudinal metabotype inter-correlation median = −0.0012). This observation was replicated in the TwinsUK study based on 83 unrelated female study participants and 203 metabolites taken at two time points that were on average 8 years apart. The median for the longitudinal metabotype intra-correlation was 0.26; for the inter-correlations, it was −0.0042 (Supplemental Figure 2a).

### Unique identification of 40 % of KORA study participants based on their metabolic profiles is possible after 7 years

**Fig. 2:**
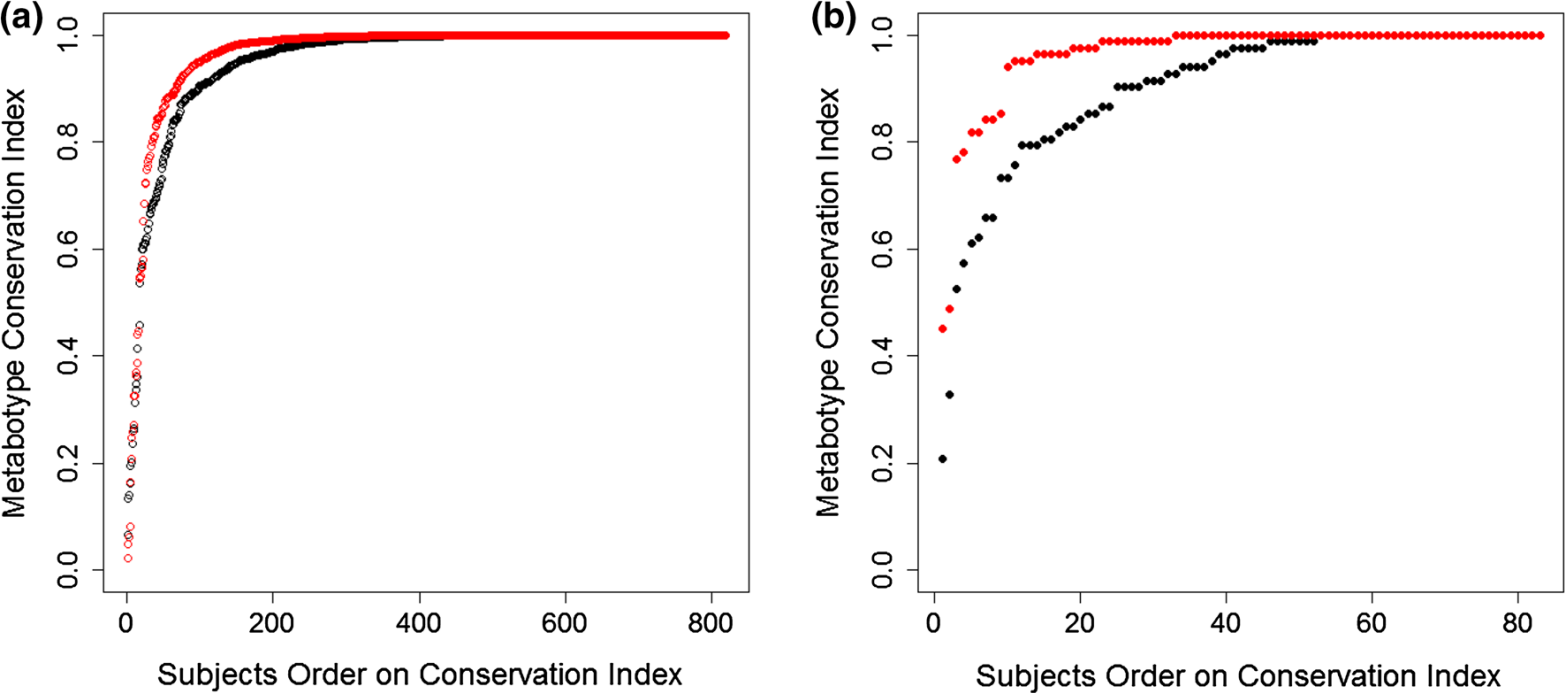
Metabotype conservation index. The conservation index of the metabotype of a study participant is defined as the relative rank of the longitudinal intra correlation of the metabolic profile of that individual compared to the longitudinal inter-correlations with the profiles of all other study participants. The conservation index is plotted in *black*, while using weighting with metabolite correlations is shown in *red*; In KORA (**a**), 40 % of the subjects have a metabotype conservation index of one, which increases to 52 % when metabolite-weighting is used. In the TwinsUK replication (**b**), the corresponding conservation index values are 37 % (*black curve*) and 61 % (*red curve*), for unweighted and weighted conservation index respectively

As a measure of human metabolic profile persistence over time, the metabotype conservation index was used. This index measures the relative rank of the individual metabotype’s longitudinal intra-correlation (correlation to self over time) within its longitudinal inter-correlation values (correlations to others over time). A conservation index value of 1 was observed for 334 out of the 818 KORA study participants, indicating that 40 % of the study participants could be uniquely identified after 7 years based on information about their metabolic profiles alone. Moreover, 95 % of the metabotypes had a conservation index above 0.7; i.e., the correlation of a metabotype in S4 to itself in F4 was ranked among the 30 % highest correlations with all other metabotypes in F4. Conversely, only 5 % of the individuals showed low conservation over time; i.e., they drastically changed their metabolic profiles over the 7-year period (black curve in Fig. 2a). This observation was also replicated in the TwinsUK study: 37 % of the participants showed a metabotype conservation index value of 1, and 95 % of all metabotypes had a conservation index above 0.57 (black curve in Fig. 2b).

### Metabolic traits are also conserved over time

We computed metabolite conservation indices and Pearson correlations for each metabolite (using correlations between individual metabolite concentrations from all individuals) for both KORA (212 metabolites) and TwinsUK (203 metabolites) (Supplemental Figure 3). Medians of metabolite longitudinal intra-correlations were 0.322 and 0.28 for KORA and TwinsUK, respectively. For 135 metabolites that were observed in both studies, we compared the metabolite conservation between the KORA and the TwinsUK studies and observed a high rank correlation (r = 0.69, *p* < 2.2 × 10^−16^) between the Pearson correlations of the metabolites from both sets. For KORA the 10 most strongly conserved metabolites are shown at the top of Table 1 and comprise 6 out of 10 sterols and steroids. With regard to other metabolite classes, lysolipids appeared among the 25 % least-conserved metabolites, and 10 out of 16 long-chain fatty acids belonged to the 50 % least-conserved metabolites. Nine out of twelve metabolites associated with valine, leucine, and isoleucine metabolism were in the top 25 % of the most-conserved metabolites. Carbohydrates were more divided, with some showing higher (e.g., 1.5 anhydroglucitol) and others lower conservation over time (e.g., pyruvate).

**Table 1:** Selected metabolites with conservation [as longitudinal intra-correlations (r)] and heritability estimates (h), restricted to metabolites with conservation or heritability greater than 0.45, which is the union of two regions of heritability ranks bounded by a ceiling of 28 and conservation ranks bounded by a ceiling of 46; ranks and difference in ranks between conservation and heritability for each metabolite are given, significant association (*p* < 0.05 after Bonferroni correction for 212 tests) of metabolites with age, gender and BMI as to a linear model (see “Sect. 2”) are indicated by ‘x’

Metabolite	r	Rank (r)	h	Rank (h)	|Rank (h) – Rank (r)|	Sex	Age	BMI
4-Androsten-3beta,17beta-diol disulfate 1	0.795	1	0.604	10	9	x	x	
Dehydroisoandrosterone sulfate	0.777	2	0.607	8	6	x	x	
4-Androsten-3beta,17beta-diol disulfate 2	0.769	3	0.582	13	10	x		x
5Alpha-androstan-3beta,17beta-diol disulfate	0.750	4	0.592	12	8	x		
Pyroglutamine	0.739	5	0.595	11	6	x		
Butyrylcarnitine	0.720	6	0.764	2	4	x		x
Thromboxane B2	0.702	7	0.606	9	2	x	x	
Androsterone sulfate	0.697	8	0.712	3	5	x		
Creatine	0.690	9	0.573	14	5	x		x
Epiandrosterone sulfate	0.683	10	0.650	4	6	x		
Alpha-hydroxyisovalerate	0.642	11	0.557	15	4	x		x
3-(4-Hydroxyphenyl)lactate	0.640	12	0.420	37	25	x		x
proline	0.610	13	0.542	17	4	x		
1,5-Anhydroglucitol	0.595	14	0.609	6	8	x		
3-Dehydrocarnitine	0.590	15	0.516	18	3	x		
Urate	0.587	16	0.609	7	9	x		x
*N*-Acetylornithine	0.551	17	0.553	16	1			
Glycine	0.546	18	0.465	24	6	x		x
Isoleucine	0.544	19	0.486	20	1	x		x
3-Carboxy-4-methyl-5-propyl-2-furanpropanoate	0.528	20	0.365	54	34			
Glutaroylcarnitine	0.523	21	0.620	5	16	x		
Isobutyrylcarnitine	0.522	22	0.464	26	4			
3-Methyl-2-oxovalerate	0.508	23	0.225	137	114	x		x
Gamma-glutamylleucine	0.505	24	0.348	59	35	x		x
Leucine	0.499	25	0.433	34	9	x		x
Betaine	0.498	26	0.417	40	14	x		
4-Vinylphenol sulfate	0.497	27	0.334	67	40	x	x	
2-Methylbutyroylcarnitine	0.491	28	0.318	77	49	x		x
Octanoylcarnitine	0.489	29	0.474	21	8			
Isovalerylcarnitine	0.486	30	0.472	22	8	x		x
*C*-Glycosyltryptophan	0.481	31	0.465	25	6		x	
Hexanoylcarnitine	0.474	32	0.492	19	13			
Kynurenine	0.473	33	0.433	33	0		x	x
4-Methyl-2-oxopentanoate	0.472	34	0.139	175	141	x		
Serotonin	0.468	35	0.331	69	34			x
Valine	0.468	36	0.412	43	7	x		x
*Cis*-4-decenoyl carnitine	0.464	37	0.436	32	5			
Erythronate	0.463	38	0.311	81	43		x	
2-Hydroxybutyrate	0.463	39	0.346	60	21	x		x
*p*-Cresol sulfate	0.463	40	0.367	51	11			
Gamma-glutamylvaline	0.459	41	0.244	125	84	x		x
Phenylacetylglutamine	0.458	42	0.332	68	26			
7-Alpha-hydroxy-3-oxo-4-cholestenoate	0.456	43	0.298	92	49	x		x
Decanoylcarnitine	0.455	44	0.416	41	3			
Docosahexaenoate	0.453	45	0.322	74	29			
Citrate	0.451	46	0.386	49	3		x	
Citrulline	0.444	47	0.471	23	24			x
Succinylcarnitine	0.418	58	0.450	28	30			x
Carnitine	0.380	73	0.453	27	46			
Homostachydrine	0.315	109	0.868	1	108		x	

The complete dataset with *p* values and beta-estimates is available as Supplemental Table 1

### Weighting metabotype correlation using metabolite conservation increases the uniquely identifiable fraction in KORA to 52 %

We hypothesized that metabolites that show a higher conservation over time also carry more information regarding an individual’s metabotype. Figure 1b shows the distributions of weighted longitudinal intra- and inter-correlations between the metabotypes of KORA at S4 and F4, where weights are the longitudinal intra-correlations of metabolites (replication for the TwinsUK set is presented in Supplemental Figure 2b). The weighting increased the median of the metabotype intra-correlations from 0.35 to 0.58 for the KORA set and from 0.26 to 0.53 for the TwinsUK set. Extending this weighting scheme to the conservation index (see Sect. 2), we observed a 30 % increase in the number of individuals who could be uniquely identified based on their metabolite profiles (from 40 % to 52 %; red curve in Fig. 2a). On an individual basis, 43 % of the participants showed an increased conservation index while only 22 % had a decreased index under this weighting procedure. For 95 % of the individuals, the metabotype conservation index was larger than 0.83, compared to 0.7 without weighting. In the TwinsUK replication study, the fraction of uniquely identifiable individuals increased from 37 to 64 % after weighting, with 95 % of all individuals having a conservation value over 0.78 (red curve in Fig. 2b).

### Individuals who display a strong change in their metabotype over time are not different from the general population

**Fig. 3:**
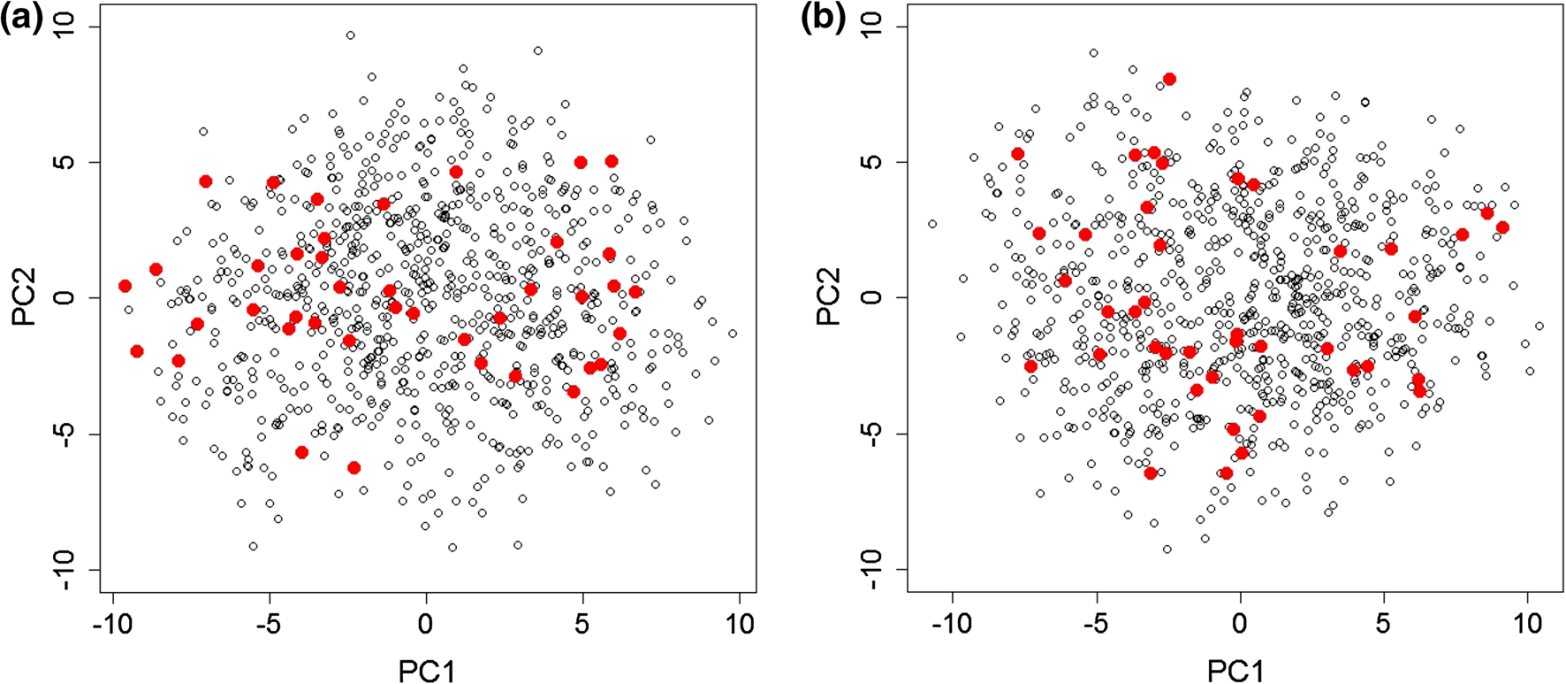
PCA of KORA S4 (**a**) and F4 (**b**) shows the 5 % least conserved individual metabotypes (*red dots*) after weighting metabotype conservation index using metabolite correlations. The least conserved metabotypes do not show a different behavior than the rest of the data, thus using both time points S4 and F4 with the conservation index is the method for identifying those least conserved ones

About 5 % of all individuals showed low longitudinal metabotype conservation (Fig. 2). To investigate whether these individuals represented outliers with extreme metabotypes, we conducted PCA. Figure 3 shows the first two dimensions of the PCA for the KORA S4 and F4 datasets, respectively. The 5 % of the least-conserved metabotypes were within the data distribution of all other individuals and thus not different from the normal population (Fig. 3).

### Most highly conserved metabolites are also highly heritable

**Fig. 4:**
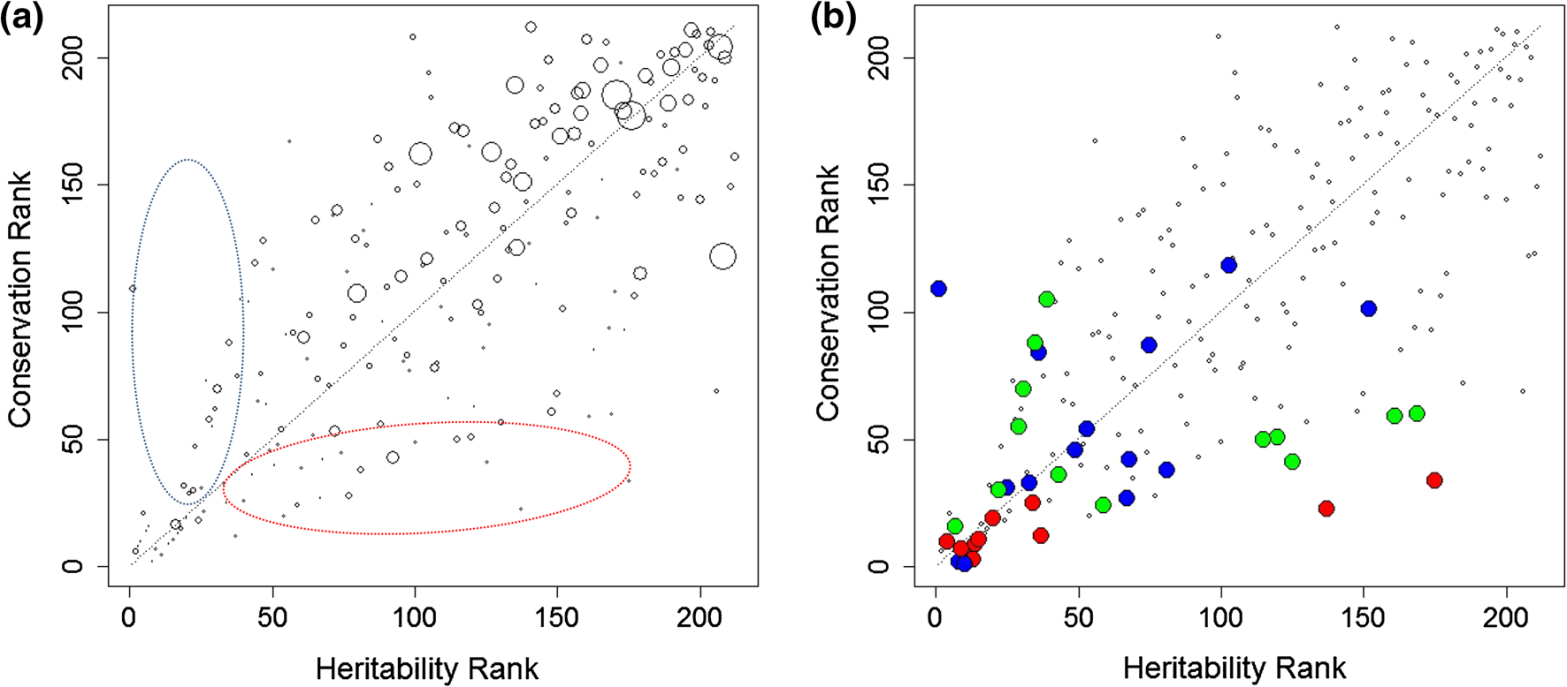
Heritability of metabolic traits compared to their conservation between two time points. **a** Marker size is proportional to the variance of technical replicates compared to their mean (RSD), and showing more heritable than conserved region in the blue ellipse area, and more conserved than heritable region in the red ellipse area. **b** The 15 most strongly associated metabolites with gender (*red*), age (*blue*), and BMI (*green*) (see Supplemental Table 2 for metabolite names)

We expected conservation of individual metabotypes to be influenced by genetic factors and hence partially heritable. To compare metabolite conservation to metabolite heritability, heritability estimates were obtained from an independent TwinsUK heritability study of over 6,000 twins (Shin et al., submitted). Figure 4 shows a cross-plot of heritability ranks and conservation ranks for all metabolites. The rank correlation between heritability and conservation was 0.74 (*p* < 2.2 × 10^−16^). Table 1 presents metabolite heritability estimates, correlation values, and respective ranks for a selected set of metabolites (full dataset, Supplemental Table 1).

### Gender, age, and BMI are associated with metabolite heritability and conservation

Gender is a conserved phenotype while BMI might change slightly over time. On the other hand, age increases identically for all individuals. Metabolites that are biomarkers for these phenotypes should thus display a higher-than-average conservation over time. To identify metabolites strongly associated with these phenotypes in the present study, linear regressions of metabolite concentrations to gender, age, and BMI were calculated (using data from the KORA S4 dataset). For each phenotype, metabolites significantly regressing (*p* < 0.05/212) and non-significantly regressing were compared for their heritability and conservation ranks, using the Wilcoxon rank test. Metabolites significantly associating with gender were also more significantly associated with high conservation ranks (*p* = 4.7 × 10^−9^) and with high heritability ranks (*p* = 7.1 × 10^−5^). Age-related metabolites also correlated with high conservation (*p* = 6.9 × 10^−4^) and with high heritability (*p* = 3.8 × 10^−4^), as did BMI (*p* = 4.6 × 10^−6^ and 2.4 × 10^−3^, for high conservation and high heritability, respectively). The 15 metabolites that associated most strongly with each phenotype are highlighted in Fig. 4b and Supplemental Table 2.

## Discussion

### Metabotype conservation

Metabolite Pearson correlations (intra-correlations between two time points) were used in this analysis to calculate a weighted metabotype conservation index as described in Sect. 2. Weighting of the metabotype correlations with the conservation of the individual metabolites over time was motivated by the observation that not all metabolites may be equally informative to identify individuals with drastic changes in their metabotype over time. For instance, variability can result from a stronger dependency on varying lifestyle factors (e.g., metabolites from diet) but also from lower measurement quality (higher RSD; see Fig. 4a). The impact of metabolite conservation on the metabotype conservation index (i.e., the 30 % increase in metabotypes with a conservation index of 1 after weighting with metabolite longitudinal intra-correlations) shows that highly conserved metabolites can be used to better distinguish individuals. This result is also supported by the findings of (Sampson et al. 2013) because some of the highly conserved metabolites from our study (Table 1) have also shown high inter-subject variability, i.e., proving to be better at discriminating individuals (see Supplemental Table 3).

Metabotypes can be divided into three categories: strictly conserved metabotypes, or those with a conservation index value of 1 (52 % of the population); highly conserved metabotypes, or those with a conservation index value in the interval [0.83,1] (43 % of the population); and least-conserved metabotypes, or those with a value in the interval [0,0.83] (5 % of the population). However, the 5 % least-conserved metabotypes presented an intriguing question regarding whether they are entirely different from the “normal” population.

To address this question, we applied PCA to KORA S4 and F4 samples to determine whether the 5 % least-conserved metabotypes are separated from the remaining metabotypes. When projected onto the first two principal components (Fig. 3), the PCA did not distinguish the least-conserved metabotypes as outliers relative to the remaining population. Other explorative techniques (such as hierarchical clustering) were used to determine if the 5 % metabotypes could be distinguished as having extreme behavior from the 95 % metabotypes when exploring each of S4 and F4 separately, as well as exploring the PCA of the mean metabotype behavior (calculated as the average of the S4 and F4 metabolic profiles for each metabotype). The results showed that no extreme behavior of the metabotypes could be determined using these techniques, either (data not shown). Thus, the two time points together rather than one time point (i.e., the longitudinal study using the conservation index) can be used to distinguish such highly changing metabotypes, once again highlighting the importance of long-term studies in detecting the abnormal behavior of metabotypes. Whether such individuals have experienced important changes in their lifestyles or developed severe diseases requires additional investigation. Results from metabotype conservation analysis further motivate the study of factors affecting metabolite behavior over time, whether because of lifestyle, environment, or genetics.

### Metabolite conservation analysis

The conservation analysis addressed the question posed by Nicholson et al. (2011) regarding the decay of metabolite stability over time. Our results indicate that even after 7 years, some metabolites remain highly conserved and contribute to increasing metabotype conservation. In exploring the conservation behavior of metabolites in different pathways, we found that the 6 steroids in the top 10 most-conserved metabolites are mostly in the androsterone pathway, which is explained by the discriminative power of gender as a “natural” individual classifier. Results from regression with gender, age, and BMI and the Wilcoxon test indicate that gender-related metabolites are more significantly associated with high conservation compared to age- and BMI-related metabolites. The conservation of gender-related metabolites has its role in increasing the metabotype conservation index, as indicated previously. This finding suggests their usability in studying the uniqueness of individual metabotypes over the long term. On the lower end of the conservation spectrum are lysolipids and the majority of the long-chain fatty acids; lysolipids are affected by nutrition but are also associated with high RSD values (>25; see Supplemental Table 1), implying that further investigation is needed for these metabolites. Examples of long-chain fatty acids that are not highly conserved include palmitate, oleate, and stearate, which are fatty acids that occur naturally in various animal and vegetable fats and oils (HMDB: http://www.hmdb.ca), and eicosenoate, which is found in a variety of plant oils. Another is margarate (heptadecanoic acid), which is known as a biological marker of long-term milk fat intake in populations with a high consumption of dairy. Food intake and lifestyle thus highly affect these long-chain fatty acids. Among the more conserved carbohydrates are 1,5-anhydroglucitol (1,5-AG), mannose, glucose, lactate, and erythronate. Because 1,5-AG is a known biomarker for short-term glycemic control (Buse et al. 2003), it is thus expected to be stable over time. It also shows a higher stability than glucose, which makes it even a stronger biomarker for diabetes. Urate appears in the top-conserved metabolites and is known to be a biomarker for Parkinson’s disease (Cipriani et al. 2010). With this overview, we have provided an example of using these results to identify metabolites that can be potentially used as biomarkers because of their stability.

Carbohydrates falling in the low-conserved region are pyruvate, erythrose, glycerate, and fructose, likely because of their high technical variance with RSD values above 25. Pyruvate showed a very low conservation compared to the more highly conserved glucose and lactate, which are in the same glycolysis pathway, and thus the RSD might explain this contradiction (Supplemental Table 1 shows annotation with pathways and superpathways together with correlation and heritability ranks of metabolites). This finding also suggests that low-conserved metabolites should be avoided when studying a metabolic disorder over time because their change with time arises from their instability rather than from an effect of the disorder’s metabolic influence.

To detect whether the conservation of some metabolites is affected by a higher conservation in one sex than the other, males and females were separated and the intra-correlations of metabolites calculated for both sexes separately, but we found no significant variation between the values obtained for each sex (Supplemental Figure 4).

### Heritability versus conservation study

Deviation between heritability ranks and conservation ranks can be used to identify metabolites that may be conserved as a result of dietary patterns or lifestyle from those that are more conserved because of their genotype association. We ordered metabolites in a descending order based on the absolute difference in heritability and conservation ranks. With this approach, taking 100 as the lower bound of absolute difference, two groups of metabolites are at the top. The first group consists of metabolites that are more conserved than heritable (they appear below the diagonal, and more towards the lower right corner of heritability graph in Fig. 4), which includes 4-methyl-2-oxopentanoate, 3-methyl-2-oxobutyrate, 3-methyl-2-oxovalerate from valine, leucine, and isoleucine metabolism, methyl palmitate from fatty acid metabolism, and glucose and lactate from the glycolysis pathway. The second group consists of metabolites that are more heritable than conserved (they appear above the diagonal and towards the upper left corner of heritability graph in Fig. 4), and these include theobromine, glycerate, and homostachydrine. Supplemental Table 2 highlights the significance of association of some of these metabolites with gender, age, and BMI. Glucose and lactate are examples of metabolites that are only moderately conserved (with conservation ranks of 59 and 60 and heritability ranks of 161 and 169, respectively), in contrast to 1,5-anhydroglucitol, which shows a high heritability and a high conservation, and pyruvate, which shows least heritability and conservation. From the second group of metabolites, those that are more heritable than conserved, glycerate significantly regressed with gender, and homostachydrine significantly regressed with age. Homostachydrine is a food compound found in citrus fruits, and citrus fruit intake undergoes both seasonal and daily variations in Germany. Because the KORA surveys were conducted over periods that are longer than 1 year and participants were enrolled randomly with respect to season, low correlation between the availability of citrus fruits to individual participants between S4 and F4 is to be expected, which may explain the lack of conservation of homostachydrine levels between S4 and F4 despite its high heritability.

Other groups of metabolites show high heritability and high conservation or low heritability and low conservation. The first group of metabolites appears in Table 1 and is significantly associated with gender, as also confirmed with results of regression to gender, age, and BMI. The other group is near the lysolipids region (upper right corner of heritability graph in Fig. 4a) and where metabolites are also associated with high RSD values (Fig. 4a). Pathway and subpathway annotation of metabolites on the heritability graph is given in Supplemental Table 1.

Results from associating heritability with conservation reveal variability among metabolites and relate it to the biological background. Along with the results of the effect of conservation on distinguishing metabotypes, these findings can be used to distinguish disorder-related phenotypes and characterize them as arising from heritability or lifestyle. Disorder-related metabolites can also be used in prediction of abnormalities in longitudinal studies.

### Limitations of the present study

Although the conservation of metabotypes confirms earlier findings from comparatively short-term studies and metabolite conservation shows results consistent with stable phenotypes (such as gender), several limitations of this study should be kept in mind. Some variation may have resulted from laboratory/technical errors associated with sample storage and the variation in the time of day at which the samples were collected at each time point. Other influences that might be limiting include the stability of serum between extraction and metabolomics analysis and variations attributable to the fasting behavior of the participants.

We use simple Pearson correlation between time points, neglecting possible influences of age, gender, and BMI on the correlation values of metabolites. However, a linear regression model that corrects for those factors was also used to calculate the correlation of metabolites and the resulting conservation index in order to evaluate the effect of these covariates. Using this more complex model did not substantially change our main results, as presented in Supplemental Figure 5.

The TwinsUK cohort presented varying time differences among participants (see Supplemental Figure 1) on the metabotype conservation. We therefore only used it for replication. It would have been interesting to study the impact of the time difference on metabolite and metabotype conservation. However, KORA involved only a fixed time difference between the two time points (i.e., 7 years), while the number of participants in the TwinsUK cohort was too low to expect statistically significant results from such an analysis, which thus was not done.

## Conclusion

We studied the long-term conservation of human individual metabolic profiles over 7 years, an essential step for extrapolation from short-term studies. We also analyzed metabolite conservation and identified poorly and highly conserved examples. More than half of the study participants could be uniquely identified after 7 years for both the KORA and TwinsUK cohorts, based on their metabotype conservation index. Highly conserved metabolites increased this uniqueness. Heritability and the 7-year conservation of metabolites were highly correlated, and the two measures together revealed variations in metabolite behavior. Metabolites that showed extremely high conservation compared to heritability or vice versa were explored for biological relevance. Results confirm the long-term conservation of individuality of metabotypes, further increasing the possibility of using metabolomics as a surrogate for understanding the systems biology underlying normal and diseased phenotypes. Metabolites reported here may be investigated as potential long-term biomarkers to detect normality/abnormality of changes in human metabolic profiles. They also stand as a reference when studying long-term changes in a metabolic disorder and to identify whether changes are the result of metabolite or disorder instability over time. The characterization of metabolites based on heritability and conservation will also be useful in understanding disease pathways and interpreting clinical studies.

## Electronic supplementary material

Below is the link to the electronic supplementary material.

Supplemental Figure 1: Ranges of time differences between the two time points in the TwinsUK data set. Mean is 8 years, 25 % quantile is 6 years, and 75 % quantile is 10 years. (PPTX 76 kb)

Supplemental Figure 2: Metabotype pairwise longitudinal inter correlations versus intra correlations distributions between TwinsUK two time points. (a) Pearson correlation of the metabolite levels between two time points for the same individual, or intra-correlations (median is 0.26 (red histogram)) and for pairwise inter correlations (median is -0.00422 (blue histogram)). (b) as in (a), but using metabolite correlations as weights to metabotype correlations (medians are 0.53 for intra-correlations (red) and -0.0047 for pairwise inter-correlations (blue)). (PPTX 187 kb)

Supplemental Figure 3: Metabolite correlation and metabolite conservation index. Distribution of the Pearson correlation coefficients for the correlation of metabolites between the two study time points for KORA (a) (212 metabolites) and TwinsUK (b), (203 metabolites); the metabolite conservation index for KORA (c) and TwinsUK (d). (PPTX 195 kb)

Supplemental Figure 4: Scatterplot of Pearson correlation of metabolites computed on the male and female subgroups in KORA alone. (PPTX 113 kb)

Supplemental Figure 5: Metabolite conservation index computed using a simple Pearson correlation (black) and using a linear model including age, gender and BMI as covariates (red). (PPTX 91 kb)

Supplemental Table 1: Excel file with the complete data (see Table 1), incl. metabolite identifiers, m/z, retention time, RSD information. (XLSX 73 kb)

Supplemental Table 2 (DOCX 28 kb)

Supplemental Table 3 (DOCX 19 kb)

## References

[CR1] Amara AW, Standaert DG (2013). Metabolomics and the search for biomarkers in Parkinson’s disease. Movement Disorders.

[CR2] Andrew T, Hart DJ, Snieder H, de Lange M, Spector TD, MacGregor AJ (2001). Are twins and singletons comparable? A study of disease-related and lifestyle characteristics in adult women. Twin Research.

[CR3] Assfalg M, Bertini I, Colangiuli D, Luchinat C, Schafer H, Schutz B (2008). Evidence of different metabolic phenotypes in humans. Proceedings of the National Academy of Sciences USA.

[CR4] Bernini P, Bertini I, Luchinat C, Nepi S, Saccenti E, Schafer H (2009). Individual human phenotypes in metabolic space and time. Journal of Proteome Research.

[CR5] Boker, S., Neale, M., Maes, H., Wilde, M., Spiegel, M., Brick, T., et al. (2011). OpenMx: An open source extended structural equation modeling framework. *Psychometrika,**76*(2), 306–317. doi:10.1007/s11336-010-9200-6. 10.1007/s11336-010-9200-6 PMC3525063 23258944

[CR6] Bollard ME, Stanley EG, Lindon JC, Nicholson JK, Holmes E (2005). NMR-based metabonomic approaches for evaluating physiological influences on biofluid composition. NMR in Biomedicine.

[CR7] Buse JB, Freeman JL, Edelman SV, Jovanovic L, McGill JB (2003). Serum 1,5-anhydroglucitol (GlycoMark): A short-term glycemic marker. Diabetes Technology and Therapeutics.

[CR8] Cipriani S, Chen X, Schwarzschild MA (2010). Urate: A novel biomarker of Parkinson’s disease risk, diagnosis and prognosis. Biomarkers in Medicine.

[CR9] Corona G, Rizzolio F, Giordano A, Toffoli G (2012). Pharmaco-metabolomics: an emerging “omics” tool for the personalization of anticancer treatments and identification of new valuable therapeutic targets. Journal of Cellular Physiology.

[CR10] Dallmann R, Viola AU, Tarokh L, Cajochen C, Brown SA (2012). The human circadian metabolome. Proceedings National Academy of Sciences USA.

[CR11] Evans AM, DeHaven CD, Barrett T, Mitchell M, Milgram E (2009). Integrated, nontargeted ultrahigh performance liquid chromatography/electrospray ionization tandem mass spectrometry platform for the identification and relative quantification of the small-molecule complement of biological systems. Analytical Chemistry.

[CR12] Fave G, Beckmann M, Lloyd AJ, Zhou S, Harold G, Lin W (2011). Development and validation of a standardized protocol to monitor human dietary exposure by metabolite fingerprinting of urine samples. Metabolomics.

[CR13] Fiehn O (2002). Metabolomics—The link between genotypes and phenotypes. Plant Molecular Biology.

[CR14] Gavaghan CL, Holmes E, Lenz E, Wilson ID, Nicholson JK (2000). An NMR-based metabonomic approach to investigate the biochemical consequences of genetic strain differences: Application to the C57BL10J and Alpk:ApfCD mouse. FEBS Letters.

[CR15] German JB, Bauman DE, Burrin DG, Failla ML, Freake HC, King JC (2004). Metabolomics in the opening decade of the 21st century: Building the roads to individualized health. Journal of Nutrition.

[CR16] Griffin JL, Shockcor JP (2004). Metabolic profiles of cancer cells. Nature Reviews Cancer.

[CR17] Gu H, Pan Z, Xi B, Hainline BE, Shanaiah N, Asiago V (2009). 1H NMR metabolomics study of age profiling in children. NMR in Biomedicine.

[CR18] Halama A, Riesen N, Moller G, Hrabe de Angelis M, Adamski J (2013). Identification of biomarkers for apoptosis in cancer cell lines using metabolomics: Tools for individualized medicine. Journal of Internal Medicine.

[CR19] Heinzmann SS, Merrifield CA, Rezzi S, Kochhar S, Lindon JC, Holmes E (2012). Stability and robustness of human metabolic phenotypes in response to sequential food challenges. Journal of Proteome Research.

[CR20] Holle R, Happich M, Lowel H, Wichmann HE (2005). KORA—A research platform for population based health research. Gesundheitswesen.

[CR21] Holmes E, Loo RL, Stamler J, Bictash M, Yap IK, Chan Q (2008). Human metabolic phenotype diversity and its association with diet and blood pressure. Nature.

[CR22] Holmes E, Wilson ID, Nicholson JK (2008). Metabolic phenotyping in health and disease. Cell.

[CR23] Kaddurah-Daouk R, Kristal BS, Weinshilboum RM (2008). Metabolomics: A global biochemical approach to drug response and disease. Annual Review of Pharmacology and Toxicology.

[CR24] Kettunen J, Tukiainen T, Sarin AP, Ortega-Alonso A, Tikkanen E, Lyytikainen LP (2012). Genome-wide association study identifies multiple loci influencing human serum metabolite levels. Nature Genetics.

[CR25] Kochhar S, Jacobs DM, Ramadan Z, Berruex F, Fuerholz A, Fay LB (2006). Probing gender-specific metabolism differences in humans by nuclear magnetic resonance-based metabonomics. Analytical Biochemistry.

[CR26] Krug S, Kastenmuller G, Stuckler F, Rist MJ, Skurk T, Sailer M (2012). The dynamic range of the human metabolome revealed by challenges. The Faseb Journal.

[CR27] Langley, R. J., Tsalik, E. L., van Velkinburgh, J. C., Glickman, S. W., Rice, B. J., Wang, C., et al. (2013). An integrated clinico-metabolomic model improves prediction of death in sepsis. *Science Translational Medicine, 5*(195), 195ra195. doi:10.1126/scitranslmed.3005893. 10.1126/scitranslmed.3005893 PMC3924586 23884467

[CR28] Lenz EM, Bright J, Wilson ID, Morgan SR, Nash AF (2003). A 1H NMR-based metabonomic study of urine and plasma samples obtained from healthy human subjects. Journal of Pharmaceutical and Biomedical Analysis.

[CR29] Lindon JC, Holmes E, Nicholson JK (2006). Metabonomics techniques and applications to pharmaceutical research and development. Pharmaceutical Research.

[CR30] Martinez-Lozano Sinues P, Kohler M, Zenobi R (2013). Human breath analysis may support the existence of individual metabolic phenotypes. PLoS ONE.

[CR31] Menni C, Fauman E, Erte I, Perry JR, Kastenmuller G, Shin SY (2013). Biomarkers for type 2 diabetes and impaired fasting glucose using a non-targeted metabolomics approach. Diabetes.

[CR32] Nicholson G, Rantalainen M, Maher AD, Li JV, Malmodin D, Ahmadi KR (2011). Human metabolic profiles are stably controlled by genetic and environmental variation. Molecular System Biology.

[CR33] Rasmussen L, Savorani F, Larsen T, Dragsted L, Astrup A, Engelsen S (2011). Standardization of factors that influence human urine metabolomics. Metabolomics.

[CR34] Rathmann W, Haastert B, Icks A, Lowel H, Meisinger C, Holle R (2003). High prevalence of undiagnosed diabetes mellitus in Southern Germany: Target populations for efficient screening. The KORA survey 2000. Diabetologia.

[CR35] Sampson JN, Boca SM, Shu XO, Stolzenberg-Solomon RZ, Matthews CE, Hsing AW (2013). Metabolomics in epidemiology: Sources of variability in metabolite measurements and implications. Cancer Epidemiology Biomarkers and Prevention.

[CR36] Saude E, Adamko D, Rowe B, Marrie T, Sykes B (2007). Variation of metabolites in normal human urine. Metabolomics.

[CR37] Suhre K, Meisinger C, Doring A, Altmaier E, Belcredi P, Gieger C (2010). Metabolic footprint of diabetes: A multiplatform metabolomics study in an epidemiological setting. PLoS ONE.

[CR38] Suhre K, Shin SY, Petersen AK, Mohney RP, Meredith D, Wagele B (2011). Human metabolic individuality in biomedical and pharmaceutical research. Nature.

[CR39] Suhre K, Shin SY, Petersen AK, Mohney RP, Meredith D, Wagele B (2011). Human metabolic individuality in biomedical and pharmaceutical research. Nature.

[CR40] Wallace M, Hashim YZ, Wingfield M, Culliton M, McAuliffe F, Gibney MJ (2010). Effects of menstrual cycle phase on metabolomic profiles in premenopausal women. Human Reproduction.

[CR41] Walsh MC, Brennan L, Malthouse JP, Roche HM, Gibney MJ (2006). Effect of acute dietary standardization on the urinary, plasma, and salivary metabolomic profiles of healthy humans. American Journal of Clinical Nutrition.

[CR42] Wang TJ, Ngo D, Psychogios N, Dejam A, Larson MG, Vasan RS (2013). 2-Aminoadipic acid is a biomarker for diabetes risk. Journal of Clinical Investigation.

[CR43] Wichmann HE, Gieger C, Illig T (2005). KORA-gen-resource for population genetics, controls and a broad spectrum of disease phenotypes. Gesundheitswesen.

[CR44] Winnike JH, Busby MG, Watkins PB, O’Connell TM (2009). Effects of a prolonged standardized diet on normalizing the human metabolome. American Journal of Clinical Nutrition.

[CR45] Zhang A, Sun H, Wang X (2012). Saliva metabolomics opens door to biomarker discovery, disease diagnosis, and treatment. Applied Biochemistry and Biotechnology.

[CR46] Zyphur MJ, Zhang Z, Barsky AP, Li W-D (2013). An ACE in the hole: Twin family models for applied behavioral genetics research. The Leadership Quarterly.

