# Supplementary figures and images for: Long term conservation of human metabolic phenotypes and link to heritability
Source: Metabolomics. 2014 Feb 26;10(5):1005–17. doi: 10.1007/s11306-014-0629-y (PMC4145193; doi:10.1007/s11306-014-0629-y)

## Slide 1
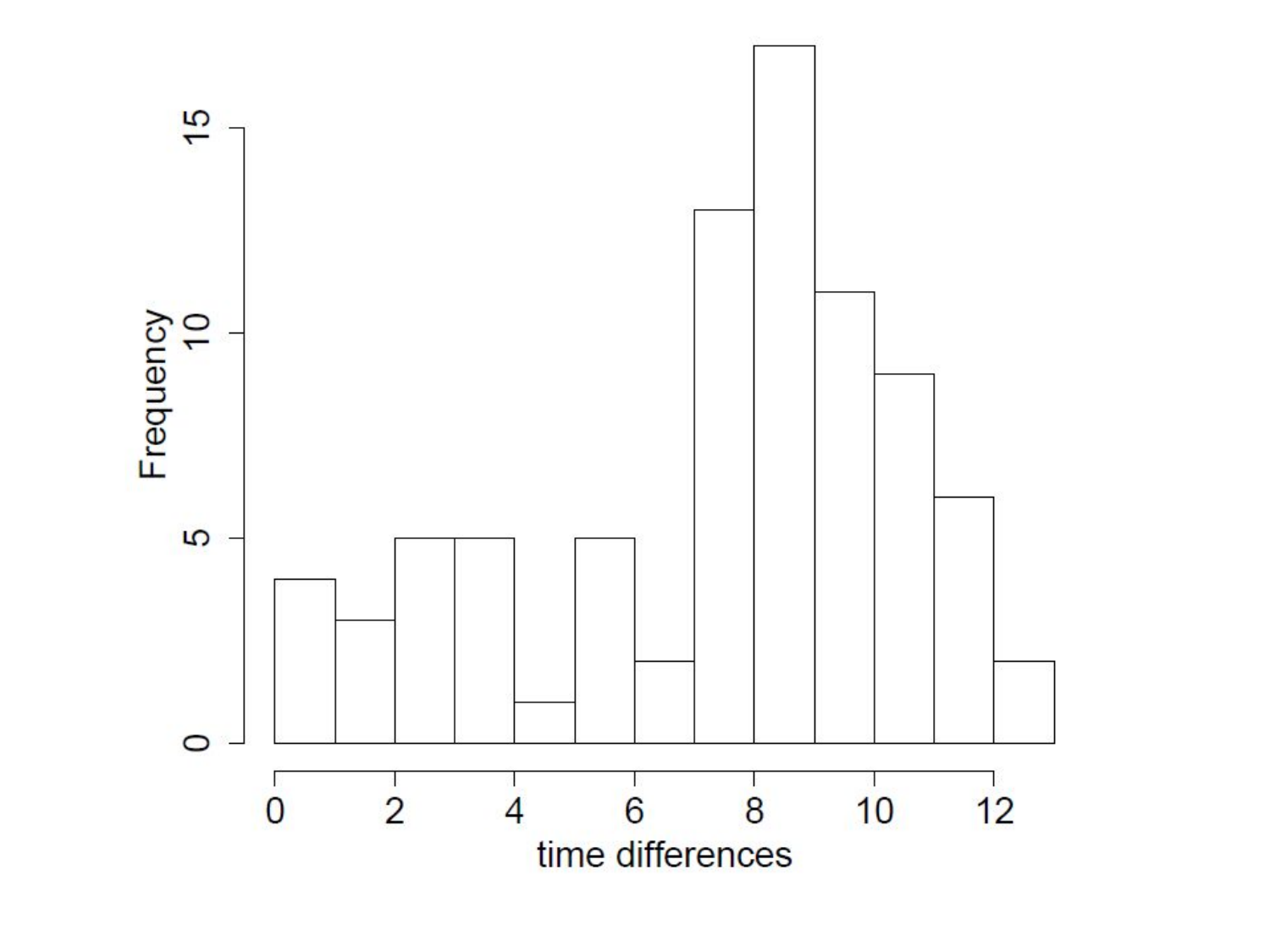

Supplement: Supplementary file 1 — Supplemental Figure 1: Ranges of time differences between the two time points in the TwinsUK data set. Mean is 8 years, 25 % quantile is 6 years, and 75 % quantile is 10 years. (PPTX 76 kb) [file 11306_2014_629_MOESM1_ESM.pptx]

## Slide 1
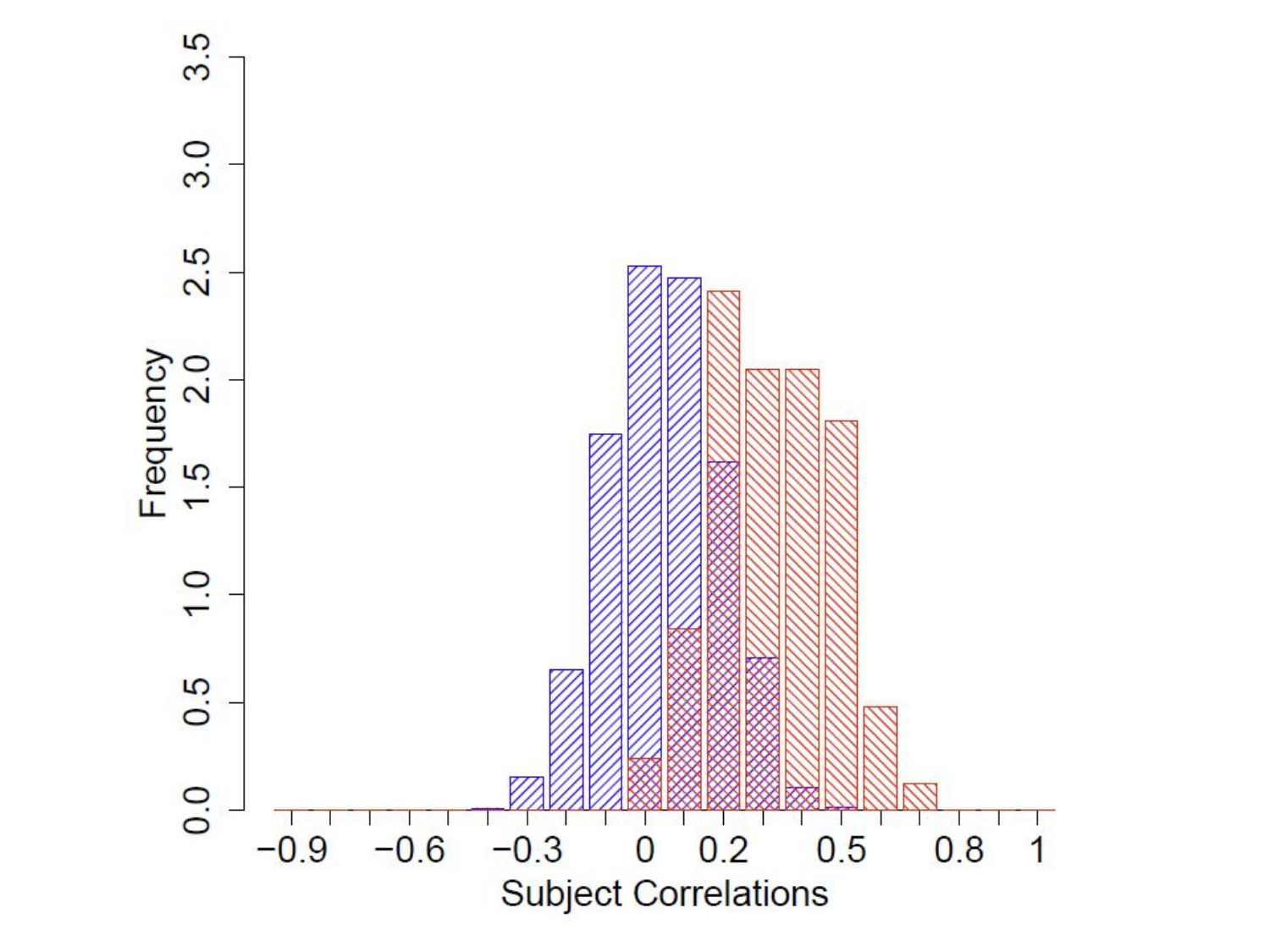

## Slide 2
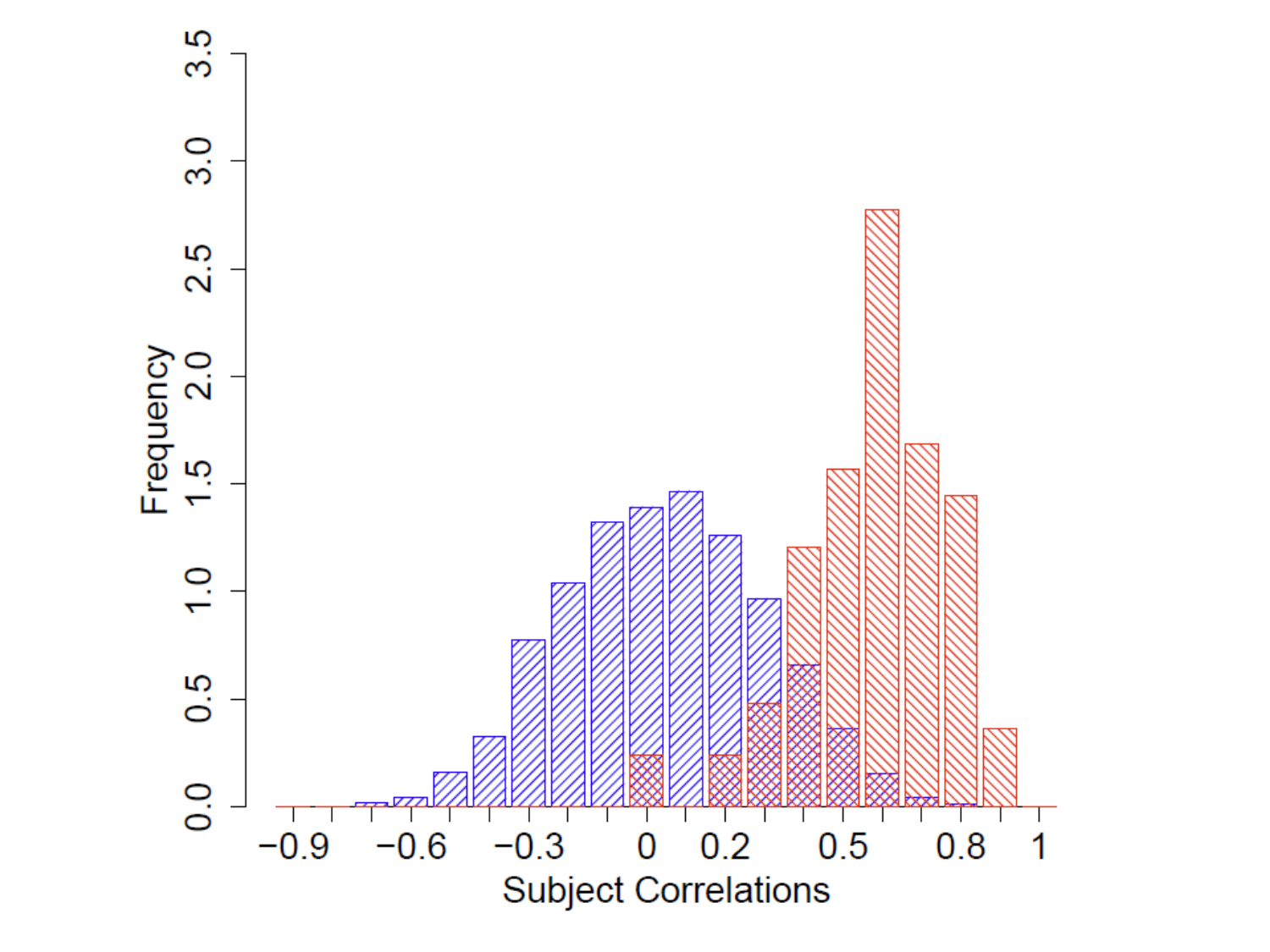

Supplement: Supplementary file 2 — Supplemental Figure 2: Metabotype pairwise longitudinal inter correlations versus intra correlations distributions between TwinsUK two time points. (a) Pearson correlation of the metabolite levels between two time points for the same individual, or intra-correlations (median is 0.26 (red histogram)) and for pairwise inter correlations (median is -0.00422 (blue histogram)). (b) as in (a), but using metabolite correlations as weights to metabotype correlations (medians are 0.53 for intra-correlations (red) and -0.0047 for pairwise inter-correlations (blue)). (PPTX 187 kb) [file 11306_2014_629_MOESM2_ESM.pptx]

## Slide 1
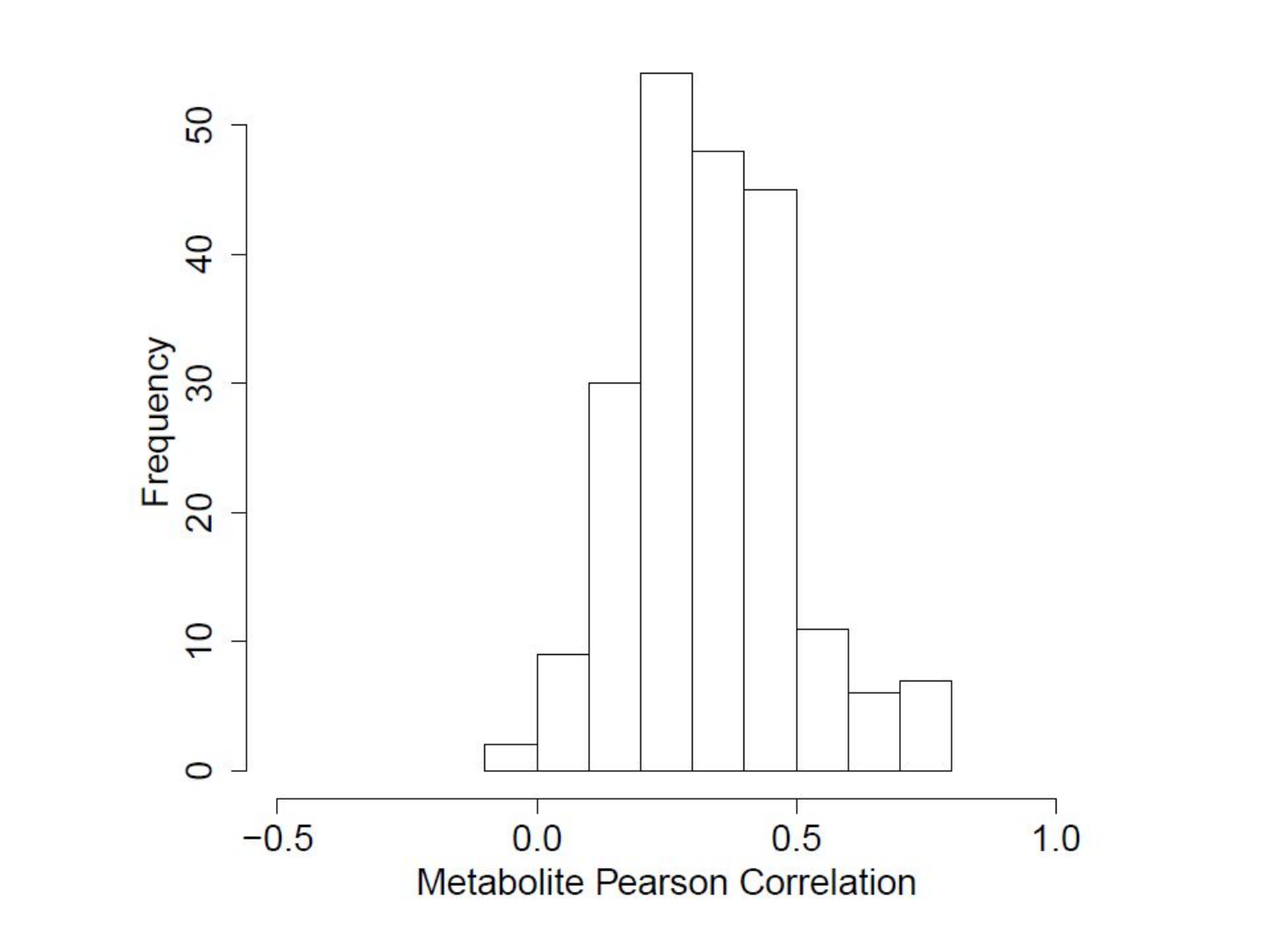

## Slide 2
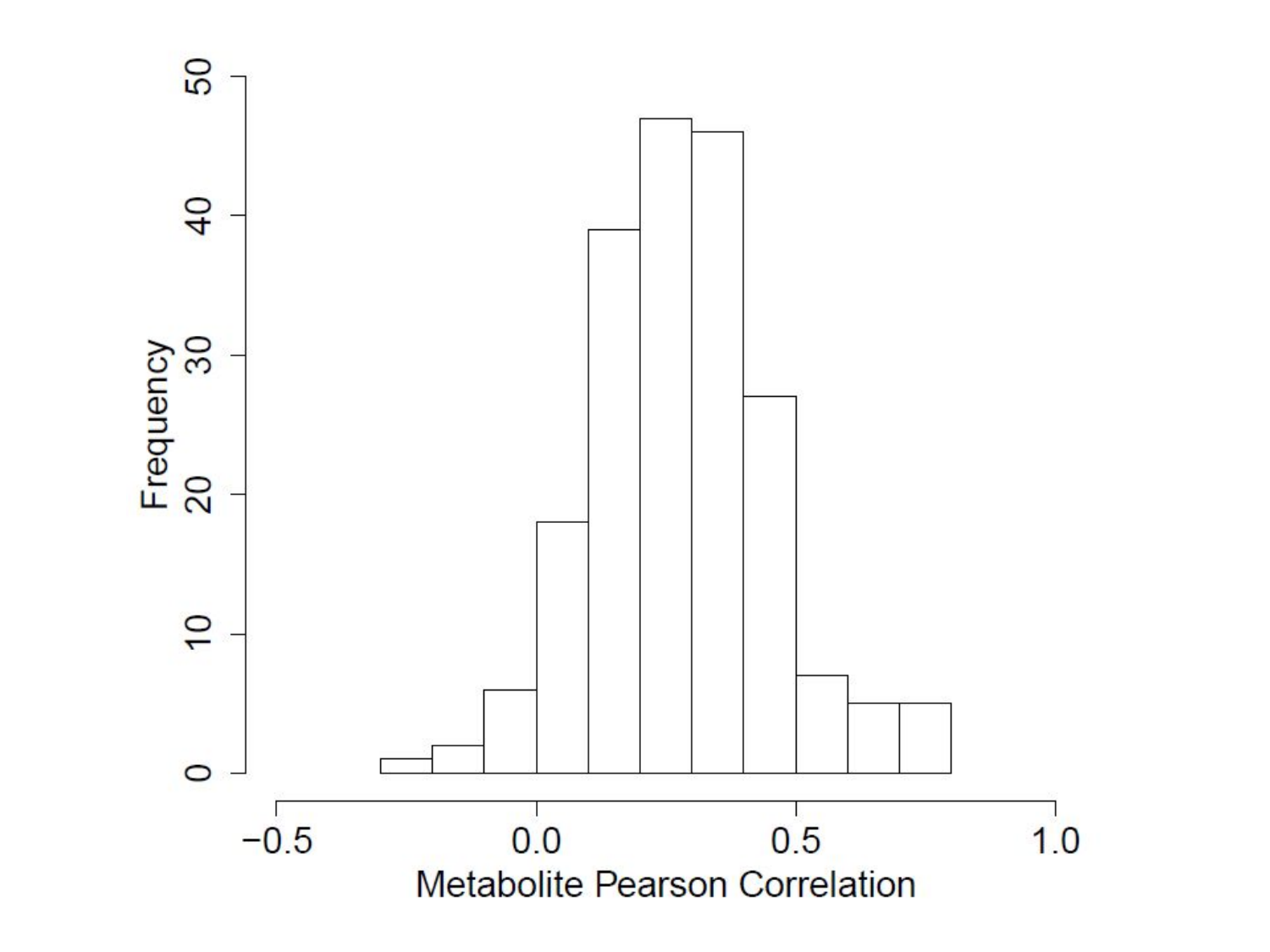

## Slide 3
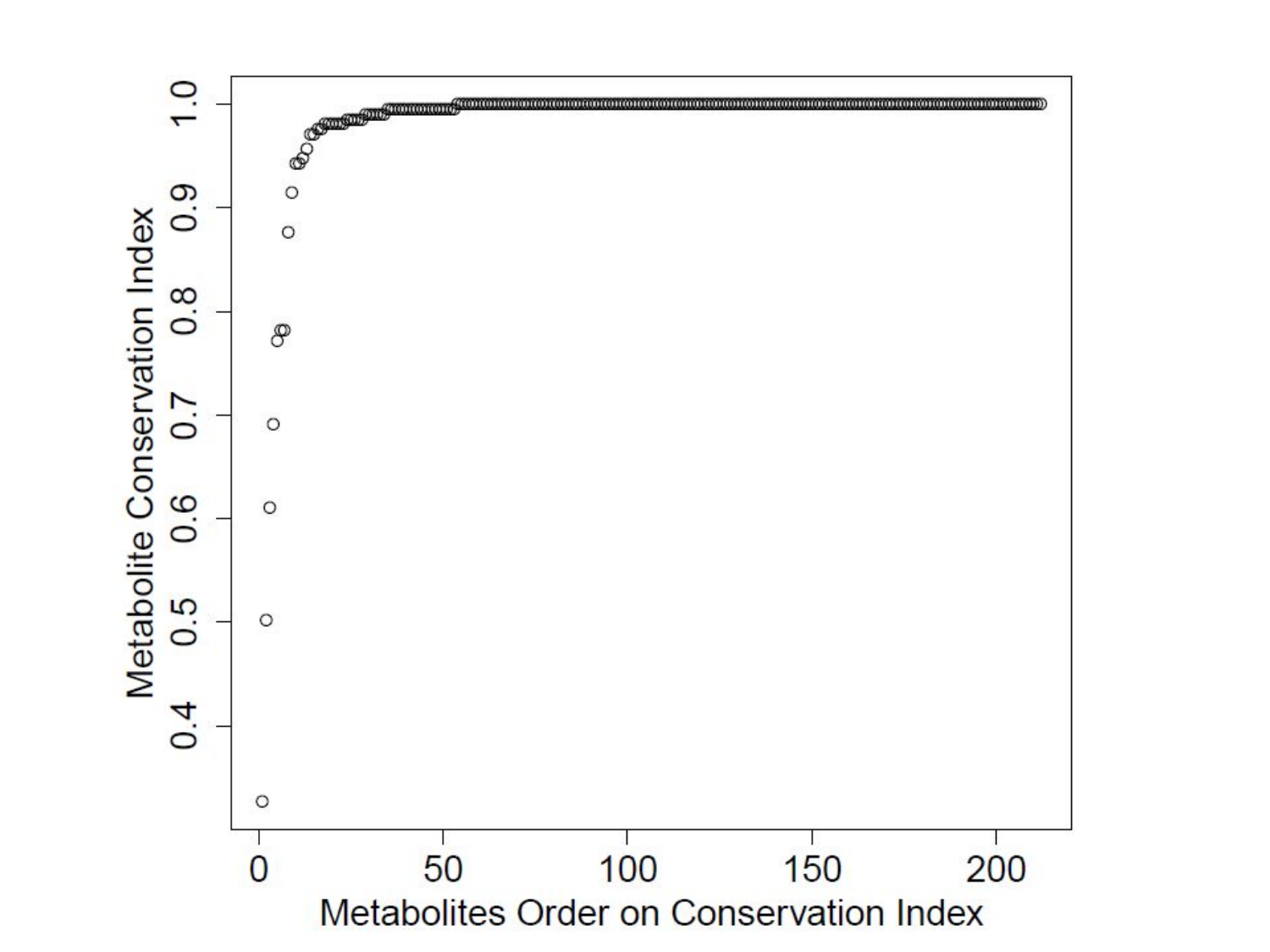

## Slide 4
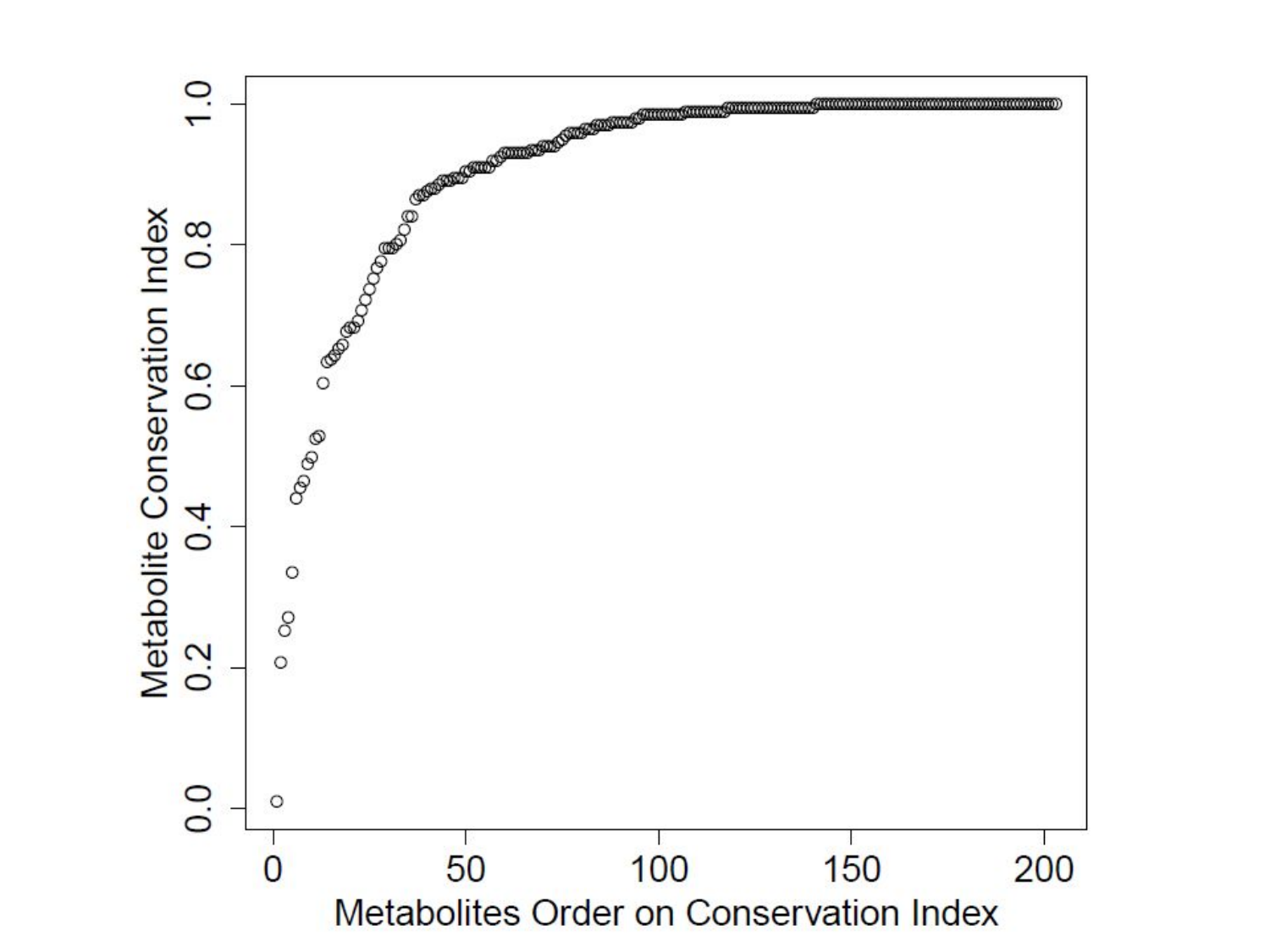

Supplement: Supplementary file 3 — Supplemental Figure 3: Metabolite correlation and metabolite conservation index. Distribution of the Pearson correlation coefficients for the correlation of metabolites between the two study time points for KORA (a) (212 metabolites) and TwinsUK (b), (203 metabolites); the metabolite conservation index for KORA (c) and TwinsUK (d). (PPTX 195 kb) [file 11306_2014_629_MOESM3_ESM.pptx]

## Slide 1
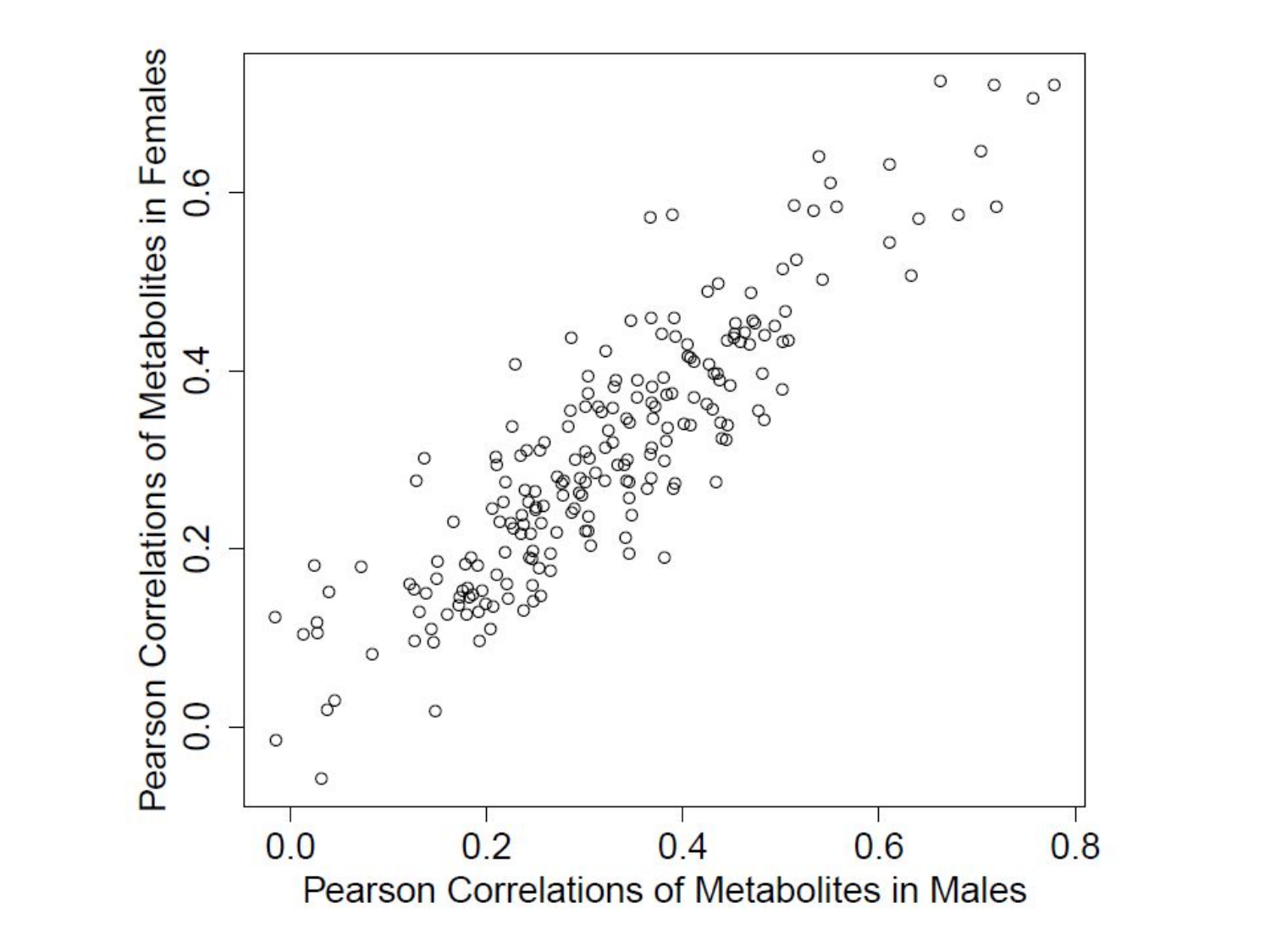

Supplement: Supplementary file 4 — Supplemental Figure 4: Scatterplot of Pearson correlation of metabolites computed on the male and female subgroups in KORA alone. (PPTX 113 kb) [file 11306_2014_629_MOESM4_ESM.pptx]

## Slide 1
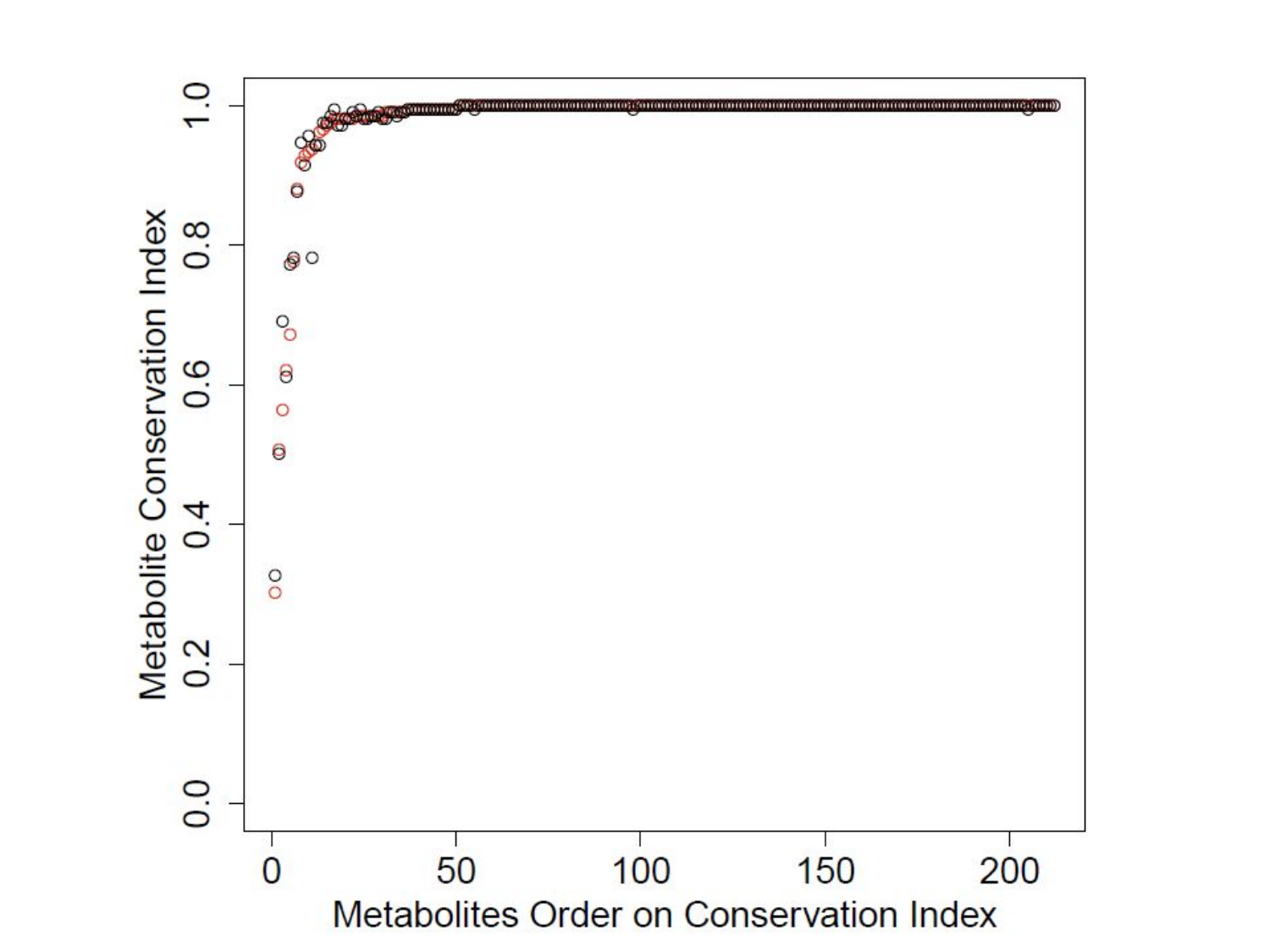

Supplement: Supplementary file 5 — Supplemental Figure 5: Metabolite conservation index computed using a simple Pearson correlation (black) and using a linear model including age, gender and BMI as covariates (red). (PPTX 91 kb) [file 11306_2014_629_MOESM5_ESM.pptx]
