# Supplementary material for: Long term conservation of human metabolic phenotypes and link to heritability
Source: Metabolomics. 2014 Feb 26;10(5):1005–17. doi: 10.1007/s11306-014-0629-y (PMC4145193; doi:10.1007/s11306-014-0629-y)
Supplement: Supplementary file 7 — Supplemental Table 2 (DOCX 28 kb) [file 11306_2014_629_MOESM7_ESM.docx]

**Table 2: Top Gender-, Age- and BMI-related metabolites**

| **Gender top related metabolites** | | | | | | | | | | | | | | | |
| --- | --- | --- | --- | --- | --- | --- | --- | --- | --- | --- | --- | --- | --- | --- | --- |
| **Rank (h)** | **Rank (r)** | **\|Rank(h) -Rank(r)\|** | **RSD** | **Gender (beta)** | **Gender (pval)** | **Age (beta)** | | **Age**  **(pval)** | | **BMI**  **(beta)** | | **BMI (pval)** | | **Name** | |
| 12 | 4 | 8 | 0 | -1.27 | 2.16E-95 | | -0.008 | | 0.17 | | 0.007 | | 0.206 | | 5alpha-androstan-3beta,17beta-diol disulfate |
| 11 | 5 | 6 | 9.3 | -1.11 | 8.81E-70 | | 0.022 | | 0.0006 | | -0.022 | | 0.0005 | | pyroglutamine |
| 10 | 1 | 9 | 0 | -0.97 | 1.08E-51 | | -0.040 | | 7.22E-09 | | 0.024 | | 0.0003 | | 4-androsten-3beta,17beta-diol disulfate 1 |
| 13 | 3 | 10 | 0 | -0.95 | 2.30E-49 | | -0.024 | | 0.0005 | | 0.029 | | 2.92E-05 | | 4-androsten-3beta,17beta-diol disulfate 2 |
| 59 | 24 | 35 | 10.9 | -0.92 | 3.59E-46 | | 0.0047 | | 0.501 | | 0.044 | | 1.55E-10 | | gamma-glutamylleucine |
| 14 | 9 | 5 | 5.4 | 0.91 | 3.75E-45 | | -0.014 | | 0.039 | | 0.033 | | 1.64E-06 | | creatine |
| 7 | 16 | 9 | 4.9 | -0.90 | 1.37E-43 | | -0.0003 | | 0.96 | | 0.043 | | 6.39E-10 | | urate |
| 37 | 12 | 25 | 8.3 | -0.89 | 1.47E-43 | | 0.0156 | | 0.028 | | 0.045 | | 8.49E-11 | | 3-(4-hydroxyphenyl)lactate |
| 15 | 11 | 4 | 9.6 | -0.86 | 5.39E-39 | | -0.009 | | 0.185 | | 0.033 | | 3.19E-06 | | alpha-hydroxyisovalerate |
| 137 | 23 | 114 | 8.3 | -0.85 | 1.11E-38 | | -0.018 | | 0.01 | | 0.033 | | 3.29E-06 | | 3-methyl-2-oxovalerate |
| 175 | 34 | 141 | 9.3 | -0.84 | 2.71E-37 | | -0.024 | | 0.001 | | 0.024 | | 0.00078 | | 4-methyl-2-oxopentanoate |
| 20 | 19 | 1 | 6 | -0.81 | 1.39E-35 | | 0.0030 | | 0.67 | | 0.046 | | 1.02E-10 | | isoleucine |
| 34 | 25 | 9 | 6 | -0.78 | 1.58E-32 | | 0.004 | | 0.55 | | 0.039 | | 7.28E-08 | | leucine |
| 4 | 10 | 6 | 5.4 | -0.77 | 4.50E-31 | | -0.023 | | 0.002 | | -0.008 | | 0.22 | | epiandrosterone sulfate |
| 9 | 7 | 2 | 7.6 | -0.74 | 1.32E-29 | | -0.053 | | 9.68E-13 | | 0.018 | | 0.01 | | thromboxane B2 |
| **Age top related metabolites** | | | | | | | | | | | | | | | |
| **Rank (h)** | **Rank (r)** | **\|Rank(h)-Rank(r)\|** | **RSD** | **Gender (beta)** | **Gender (pval)** | | **Age (beta)** | | **Age**  **(pval)** | | **BMI**  **(beta)** | | **BMI (pval)** | | **Name** |
| 8 | 2 | 6 | 4.2 | -0.69 | 3.29E-26 | | -0.05804 | | 1.48E-14 | | 0.007 | | 0.27 | | dehydroisoandrosterone sulfate |
| 9 | 7 | 2 | 7.6 | -0.74 | 1.32E-29 | | -0.05343 | | 9.68E-13 | | 0.018 | | 0.01 | | thromboxane B2 |
| 25 | 31 | 6 | 9.4 | 0.19 | 0.004 | | 0.05688 | | 1.15E-12 | | 0.013 | | 0.085 | | C-glycosyltryptophan |
| 10 | 1 | 9 | 0 | -0.97 | 1.08E-51 | | -0.04088 | | 7.22E-09 | | 0.024 | | 0.00034 | | 4-androsten-3beta,17beta-diol disulfate 1 |
| 53 | 54 | 1 | 14.6 | -0.17 | 0.01 | | 0.04642 | | 7.54E-09 | | 0.02 | | 0.0092 | | pseudouridine |
| 36 | 84 | 48 | 0 | -0.09 | 0.18 | | -0.04043 | | 6.33E-07 | | -0.006 | | 0.38 | | tryptophan betaine |
| 49 | 46 | 3 | 9.2 | 0.18 | 0.0059 | | 0.03924 | | 1.14E-06 | | -0.027 | | 0.00039 | | citrate |
| 81 | 38 | 43 | 15.9 | -0.033 | 0.62 | | 0.03811 | | 2.33E-06 | | 0.025 | | 0.001353 | | erythronate |
| 33 | 33 | 0 | 7.8 | -0.189 | 0.005 | | 0.03543 | | 8.19E-06 | | 0.041 | | 7.18E-08 | | kynurenine |
| 1 | 109 | 108 | 17 | -0.11 | 0.097 | | 0.03532 | | 1.36E-05 | | -0.020 | | 0.0085 | | homostachydrine |
| 75 | 87 | 12 | 15.1 | 0.044 | 0.51 | | 0.034284 | | 2.12E-05 | | 0.028 | | 0.00025 | | erythritol |
| 103 | 118 | 15 | 11.7 | -0.008 | 0.9 | | 0.03206 | | 8.45E-05 | | 0.003 | | 0.63 | | N-acetylalanine |
| 67 | 27 | 40 | 6.5 | -0.62 | 1.51E-20 | | -0.02926 | | 0.00015 | | -0.01 | | 0.15 | | 4-vinylphenol sulfate |
| 152 | 101 | 51 | 15.3 | -0.023 | 0.734 | | 0.03029 | | 0.00020 | | -0.005 | | 0.49 | | 4-acetamidobutanoate |
| 68 | 42 | 26 | 6.9 | 0.089 | 0.199 | | 0.02928 | | 0.00033 | | -0.003 | | 0.62 | | phenylacetylglutamine |
| **BMI top related metabolites** | | | | | | | | | | | | | | | |
| **Rank (h)** | **Rank (r)** | **\|Rank(h)-Rank(r)\|** | **RSD** | **Gender (beta)** | **Gender (pval)** | **Age (beta)** | | **Age**  **(pval)** | | **BMI**  **(beta)** | | **BMI (pval)** | | **Name** | |
| 120 | 51 | 69 | 15.7 | -0.38 | 4.69E-09 | 0.0052 | | 0.49 | | 0.064 | | 6.85E-17 | | glutamate | |
| 125 | 41 | 84 | 9.3 | -0.7 | 3.52E-27 | 0.013 | | 0.075 | | 0.055 | | 2.46E-14 | | gamma-glutamylvaline | |
| 29 | 55 | 26 | 6.1 | -0.4 | 1.92E-09 | 0.01 | | 0.17 | | 0.057 | | 6.21E-14 | | tyrosine | |
| 115 | 50 | 65 | 17.1 | -0.3 | 7.46E-06 | 0.013 | | 0.09 | | 0.055 | | 8.77E-13 | | mannose | |
| 31 | 70 | 39 | 21.9 | 0.42 | 2.22E-10 | 0.009 | | 0.21 | | -0.054 | | 1.17E-12 | | N-acetylglycine | |
| 43 | 36 | 7 | 5.9 | -0.57 | 1.53E-17 | 0.008 | | 0.28 | | 0.049 | | 4.34E-11 | | valine | |
| 37 | 12 | 25 | 8.3 | -0.89 | 1.47E-43 | 0.015 | | 0.028 | | 0.045 | | 8.49E-11 | | 3-(4-hydroxyphenyl)lactate | |
| 35 | 88 | 53 | 17.2 | -0.51 | 1.14E-14 | 0.02 | | 0.008 | | 0.049 | | 9.44E-11 | | gamma-glutamyltyrosine | |
| 20 | 19 | 1 | 6 | -0.81 | 1.39E-35 | 0.003 | | 0.67 | | 0.046 | | 1.02E-10 | | isoleucine | |
| 59 | 24 | 35 | 10.9 | -0.92 | 3.59E-46 | 0.0047 | | 0.50 | | 0.044 | | 1.55E-10 | | gamma-glutamylleucine | |
| 39 | 105 | 66 | 5.8 | -0.25 | 0.00014 | 0.0074 | | 0.34 | | 0.048 | | 4.24E-10 | | phenylalanine | |
| 7 | 16 | 9 | 4.9 | -0.90 | 1.37E-43 | -0.00034 | | 0.96 | | 0.043 | | 6.39E-10 | | urate | |
| 161 | 59 | 102 | 9.6 | -0.18 | 0.00813 | 0.008 | | 0.29 | | 0.048 | | 8.86E-10 | | glucose | |
| 169 | 60 | 109 | 8.6 | -0.13 | 0.04841 | 0.007 | | 0.37 | | 0.047 | | 2.15E-09 | | lactate | |
| 22 | 30 | 8 | 12.9 | -0.73 | 2.04E-28 | -0.0089 | | 0.22 | | 0.043 | | 3.70E-09 | | isovalerylcarnitine | |
