# Supplementary material for: Long term conservation of human metabolic phenotypes and link to heritability
Source: Metabolomics. 2014 Feb 26;10(5):1005–17. doi: 10.1007/s11306-014-0629-y (PMC4145193; doi:10.1007/s11306-014-0629-y)
Supplement: Supplementary file 8 — Supplemental Table 3 (DOCX 19 kb) [file 11306_2014_629_MOESM8_ESM.docx]

**Table 3: Metabolic Variability [Sampson et. al] compared to heritability and conservation ranks.**

| **Name** | **variability** | **Variability rank** | **Heritability rank** | **Correlation rank** | **\|Rank(h)-Rank(r)\|** |
| --- | --- | --- | --- | --- | --- |
| 1,5-Anhydroglucitol (1,5-AG) | 0.91 | 1 | 6 | 14 | 8 |
| 4-Androsten-3b,17b-diol disulfate 1 | 0.9 | 2 | 10 | 1 | 9 |
| 4-Androsten-3b,17b-diol disulfate 2 | 0.85 | 3 | 13 | 3 | 10 |
| Pyroglutamine | 0.83 | 4 | 11 | 5 | 6 |
| Androsterone sulfate | 0.82 | 5 | 3 | 8 | 5 |
| 5a-Androstan-3b,17b-diol disulfate | 0.8 | 6 | 12 | 4 | 8 |
| Epiandrosterone sulfate | 0.79 | 7 | 4 | 10 | 6 |
| a-Hydroxyisovalerate | 0.76 | 8 | 15 | 11 | 4 |
| 3-(4-Hydroxyphenyl)lactate | 0.76 | 9 | 37 | 12 | 25 |
| Urate | 0.74 | 10 | 7 | 16 | 9 |
| C-Glycosyltryptophan | 0.74 | 11 | 25 | 31 | 6 |
| Glutaroyl carnitine | 0.72 | 12 | 5 | 21 | 16 |
| Creatine | 0.72 | 13 | 14 | 9 | 5 |
| 3-Dehydrocarnitine | 0.72 | 14 | 18 | 15 | 3 |
| 1-Arachidonoylglycerophosphocholine | 0.72 | 15 | 127 | 163 | 36 |
| 2-Hydroxybutyrate (AHB) | 0.71 | 16 | 60 | 39 | 21 |
